# BCKDH: The Missing Link in Apicomplexan Mitochondrial Metabolism Is Required for Full Virulence of *Toxoplasma gondii* and *Plasmodium berghei*

**DOI:** 10.1371/journal.ppat.1004263

**Published:** 2014-07-17

**Authors:** Rebecca D. Oppenheim, Darren J. Creek, James I. Macrae, Katarzyna K. Modrzynska, Paco Pino, Julien Limenitakis, Valerie Polonais, Frank Seeber, Michael P. Barrett, Oliver Billker, Malcolm J. McConville, Dominique Soldati-Favre

**Affiliations:** 1 Department of Microbiology and Molecular Medicine, Faculty of Medicine, University of Geneva, Geneva, Switzerland; 2 Department of Biochemistry and Molecular Biology, Bio21 Molecular Science and Biotechnology Institute, University of Melbourne, Parkville, Victoria, Australia; 3 Wellcome Trust Centre for Molecular Parasitology and Glasgow Polyomics, University of Glasgow, Glasgow, United Kingdom; 4 Drug Delivery Disposition and Dynamics, Monash Institute of Pharmaceutical Sciences, Monash University, Parkville, Victoria, Australia; 5 The National Institute for Medical Research, Mill Hill, London, United Kingdom; 6 Wellcome Trust Sanger Institute, Hinxton, Cambridge, United Kingdom; 7 FG16 - Mycotic and parasitic agents and mycobacteria, Robert Koch Institute, Berlin, Germany; Washington University School of Medicine, United States of America

## Abstract

While the apicomplexan parasites *Plasmodium falciparum* and *Toxoplasma gondii* are thought to primarily depend on glycolysis for ATP synthesis, recent studies have shown that they can fully catabolize glucose in a canonical TCA cycle. However, these parasites lack a mitochondrial isoform of pyruvate dehydrogenase and the identity of the enzyme that catalyses the conversion of pyruvate to acetyl-CoA remains enigmatic. Here we demonstrate that the mitochondrial branched chain ketoacid dehydrogenase (BCKDH) complex is the missing link, functionally replacing mitochondrial PDH in both *T. gondii* and *P. berghei*. Deletion of the E1a subunit of *T. gondii* and *P. berghei* BCKDH significantly impacted on intracellular growth and virulence of both parasites. Interestingly, disruption of the *P. berghei* E1a restricted parasite development to reticulocytes only and completely prevented maturation of oocysts during mosquito transmission. Overall this study highlights the importance of the molecular adaptation of BCKDH in this important class of pathogens.

## Author Summary

The mitochondrial tricarboxylic acid (TCA) cycle is one of the core metabolic pathways of eukaryotic cells, which contributes to cellular energy generation and provision of essential intermediates for macromolecule synthesis. Apicomplexan parasites possess the complete sets of genes coding for the TCA cycle. However, they lack a key mitochondrial enzyme complex that is normally required for production of acetyl-CoA from pyruvate, allowing further oxidation of glycolytic intermediates in the TCA cycle. This study unequivocally resolves how acetyl-CoA is generated in the mitochondrion using a combination of genetic, biochemical and metabolomic approaches. Specifically, we show that *T. gondii* and *P. bergei* utilize a second mitochondrial dehydrogenase complex, BCKDH, that is normally involved in branched amino acid catabolism, to convert pyruvate to acetyl-CoA and further catabolize glucose in the TCA cycle. In *T. gondii*, loss of BCKDH leads to global defects in glucose metabolism, increased gluconeogenesis and a marked attenuation of growth in host cells and virulence in animals. In *P. bergei*, loss of BCKDH leads to a defect in parasite proliferation in mature red blood cells, although the mutant retains the capacity to proliferate within 'immature' reticulocytes, highlighting the role of host metabolism/physiology on the development of *Plasmodium* asexual stages.

## Introduction

The phylum of *Apicomplexa* comprises a large number of obligate intracellular parasites that infect organisms across the whole animal kingdom. Two important members of this phylum, *Plasmodium* spp. and *Toxoplasma gondii*, are the etiological agents of malaria and toxoplasmosis, respectively. Malaria remains one of the most significant global public health challenges (World Malaria Report 2012, www.who.int), while toxoplasmosis causes severe disease and death in immunocompromised individuals and can lead to complications in development of the foetus if contracted during pregnancy [1].

Both *Plasmodium* spp and *T. gondii* invade a range of mammalian cells and replicate within a membrane-enclosed compartment called the parasitophorous vacuole (PV). Residence within the PV provides protection from host cell defence mechanisms, while allowing the rapidly developing parasite stages to access small molecules that can diffuse freely across the PV membrane (PVM) [2], [3]. Both *T. gondii* replicative forms and *Plasmodium* blood stages were thought to rely primarily on glucose uptake and glycolysis for generation of ATP and other intermediates required for energy generation and replication [4]–[8], and to lack a canonical, pyruvate-fuelled TCA cycle. In particular, *Plasmodium*-infected erythrocytes exhibit an extraordinarily high rate of glucose uptake [9] and selective inhibitors of the *Plasmodium* hexose transporter are cytotoxic [10], [11]. Moreover, genomic and biochemical studies have shown that apicomplexan parasites target their single canonical pyruvate dehydrogenase complex (PDH) to the apicoplast, a non-photosynthetic plastid organelle involved in fatty acid biosynthesis, rather than to the mitochondrion [12]–[14]. The absence of a mitochondrial PDH complex in these parasites suggested that glycolytic pyruvate was not converted to acetyl-CoA in the mitochondrion and further catabolised through the TCA cycle [13]–[15].

**Figure 1:**
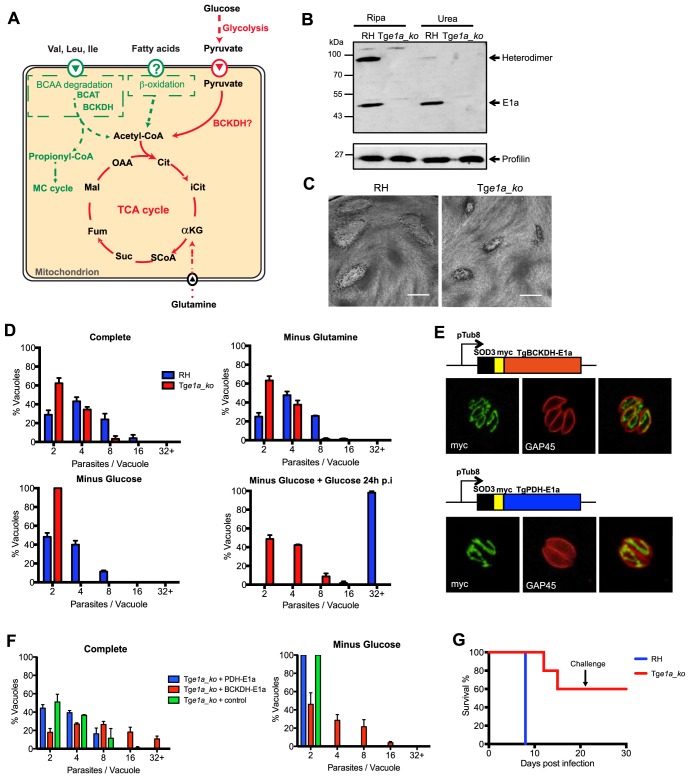
*Toxoplasma gondii* BCKDH-complex is required for normal growth and virulence. (A) Schematic representation of pathways to produce acetyl-CoA in the mitochondrion. In green are pathways specific to *T. gondii* and in red pathways common to *T. gondii* and *Plasmodium* spp. (B) Total lysates from extracellular RH*ku80_ko* (RH) and Tg*e1a_ko* tachyzoites were analysed by Western blot. Expression of BCKDH-E1a was assessed using polyclonal anti-PfBCKDH-E1a antibodies. Detection of profilin was used as loading control. (C) Plaque assays were performed by inoculating HFF monolayers with RH or Tg*e1a_ko* parasites for 7 days. Plaques were revealed by Giemsa staining of HFFs. Scale bar represents 1 mm. (D) Intracellular growth of RH (blue) and Tg*e1a_ko* (red) was assessed after 24 h in complete media, media lacking glutamine, or glucose. Following 24 h of growth in glucose-depleted environment, glucose was added back to the media and rescue of the parasite’s growth was assessed. Data are represented as means ± SD from three independent biological replicates. (E) The apicoplast targeting sequence of TgPDH-E1a (aa 1–225, ABE76506) and mitochondrial targeting sequence of TgBCKDH-E1a (aa 1–73, XP_002366588) were replaced with the mitochondrial transit peptide of the superoxide dismutase 3 (SOD3) and myc-tagged [71] to direct the expression of the fusion protein in the mitochondrion of Tg*e1a_ko* parasites for complementation (creating pTub8-SOD3mycPDHE1a and pTub8-SOD3mycBCKDHE1a respectively). Immunofluorescence assay shows localization of SOD3mycPDHE1a and SOD3mycBCKDHE1a in the single tubular mitochondrion (anti-myc (in green), anti-GAP45 (pellicle marker in red)). (F) Intracellular growth assay at 32 h post transient transfection of Tg*e1a_ko* with pTub8-SOD3mycPDHE1a, pTub8-SOD3mycBCKDHE1a and pTub8-mycNtGAP45 (negative control) in complete media or media depleted in glucose. Data are represented as means ± SD from three independent biological replicates. Only vacuoles containing parasites transiently expressing the transgene were taken into account. Over 200 vacuoles were counted per replicate. (G) CD1 mice were infected with RH (in blue) or Tg*e1a_ko* (in red) tachyzoites (∼15 parasites per mouse) and survival was assessed over 21 days. A challenge with ∼1000 wild-type RH tachyzoites was performed on mice that survived initial infection and survival followed for a further 10 days. Five mice were infected per condition.

In other organisms, lipids and branched chain amino acids (BCAA) can be catabolised in the mitochondrion to generate acetyl-CoA via pathways not dependent on PDH (Fig. 1A). However, *Plasmodium* spp. lack the enzymes needed for the β-oxidation of fatty acids and BCAA degradation. While *T. gondii* retained the enzymatic machinery necessary for β-oxidation, these parasites appear to lack a typical mitochondrial acyl-carnitine/carnitine carrier [16], [17]. Moreover, the genes coding for β-oxidation enzymes are apparently not expressed in tachyzoites, although they may be active in oocysts [18]. The possibility that *T. gondii* tachyzoites rely on host BCAA to generate mitochondrial acetyl-CoA was recently investigated, but disruption of the gene encoding the first enzyme involved in BCAA degradation, branched chain amino acid transferase (BCAT) in tachyzoites presented no phenotypic defect [19]. Together, these studies suggested that there was minimal synthesis and catabolism of acetyl-CoA in the mitochondrion.

Several studies have recently led to a reappraisal of this model of carbon metabolism in *Apicomplexa*. Firstly, detailed ^13^C-glucose and ^13^C-glutamine tracer experiments on *T. gondii* tachyzoite stages showed that carbon skeletons derived from both carbon sources were actively catabolised in a canonical TCA cycle, with the majority of pyruvate entering via acetyl-CoA. Chemical disruption of the aconitase enzyme activity, catalysing an early step in the TCA cycle, completely ablated parasite growth and infectivity in mammalian cells, indicating that the conversion of citrate to isocitrate is important for parasite growth and pathogenesis and that dysregulation of glucose catabolism in the mitochondrion is likely to be lethal [20]. Second, similar studies undertaken in *P. falciparum* indicate that glucose is further catabolised in the TCA cycle in asexual blood stages [21]–[23], and at a dramatically increased rate in sexual gametocyte stages [21]. A functional, canonical TCA cycle capable of generating reducing equivalents is likely necessary for maintenance of mitochondrial protein transport and the re-oxidation of inner membrane dehydrogenases required for pyrimidine biosynthesis [24]–[28]. The importance of an active respiratory chain in *P. falciparum* blood stages is highlighted by the sensitivity of this stage to the antimalarial drug atovaquone, which targets respiratory chain complexes [29], and by the increased expression of TCA cycle enzymes in parasites isolated from patients in endemic areas [30], [31]. Atovaquone is also known to kill the rapidly dividing tachyzoite and cyst-forming bradyzoite stages of *T. gondii* [32].

These studies suggest that the translocation of the conventional mitochondrial PDH to the apicoplast was associated with a new enzyme activity that functionally replaced PDH in regulating TCA cycle metabolism, although the identity of this enzyme remains unknown. The possibility that other mitochondrial dehydrogenases may individually or collectively fill this missing link was raised by the finding that the *P. falciparum* α-ketoglutarate dehydrogenase (α-KDH) can catalyze the conversion of pyruvate to acetyl-CoA *in vitro*, although slightly less efficiently than PDH [33]. On the other hand, Cobbold et al., proposed that the *P. falciparum* branched chain ketoacid dehydrogenase (BCKDH), the only enzyme implicated in BCAA degradation retained in the *Plasmodium* spp., may substitute for PDH based on the finding that catabolism of glucose in the TCA cycle in a *P. falciparum* PDH mutant was inhibited by oxythiamine, an inhibitor of thiamine pyrophosphate (TPP)-dependent dehydrogenases [22]. However, oxythiamine also inhibits α-KDH (and all other TPP-dependent enzymes), and the enzymatic activity of *P. falciparum* BCKDH was not tested. The identity of the enzyme(s) that link glycolysis with mitochondrial metabolism, and their functional significance in the normal growth and virulence of these parasites therefore remains an open question.

The genomes of the apicomplexan parasites that contain a functional mitochondrion encode all of the subunits of the BCKDH complex, which include the branched chain α-keto acid dehydrogenase E1 subunits (EC 1.2.4.4), the dihydrolipoyl transacylase E2 subunit (EC 2.3.1.168), and the lipoamide dehydrogenase E3 subunit (EC 1.8.1.4)–(Table S1). The eukaryotic BCKDH and PDH complexes share many structural and enzymatic properties, catalysing analogous reactions in central carbon metabolism where the initial α-ketoacid is decarboxylated by the E1 subunit - a thiamine diphosphate (TPP)-dependent heterotetramer consisting of two α subunits (E1a) and two β subunits (E1b) [34]. Given the functional similarity between these complexes, we have previously postulated that the BCKDH complex could have assumed the function of the mitochondrial PDH in the *Apicomplexa* [16].

In this study, we provide unequivocal evidence that BCKDH primarily fulfils the function of mitochondrial PDH in both *T. gondii* and *Plasmodium berghei*, a rodent model for malaria. *P. berghei* allows phenotypic evaluation under physiological conditions *in vivo* and offers the potential to interrogate the whole parasite life cycle from mosquito to mouse. We find that genetic disruption of the BCKDH-E1a subunit in these parasites leads to a block in the conversion of pyruvate to acetyl-CoA, and global changes in metabolic fluxes, as shown by metabolite profiling and comprehensive ^13^C-stable isotope labelling approaches. More importantly, the functional disruption of the BCKDH multi-enzyme complex was associated with a growth defect and reduced virulence of *T. gondii* in mice, while in *P. berghei* it resulted in strong alteration of intraerythrocytic development and severely diminished virulence in mice. In addition, the absence of BCKDH affects all the vector stages and blocks oocyst development in the mosquito, indicating that this pathway is essential for transmission of the disease.

## Results

### *Toxoplasma gondii* BCKDH is required for normal intracellular growth and virulence

Point mutations in human BCKDH-E1a are associated with complete loss of catalytic activity [34], indicating that genetic depletion of this subunit should be sufficient to abrogate BCKDH function. Deletion of the gene coding for the E1a subunit of TgBCKDH was achieved by double homologous recombination (Tg*e1a_ko*) in the RH*ku80_ko* (hereafter termed ‘RH’) background strain, which favours homologous recombination over random integration (Fig. S1A) [35], [36]. Transgenic parasites were cloned and loss of *TgBCKDHE1a* was demonstrated by genomic PCR (Fig. S1B), while absence of the protein was confirmed by Western blot using cross-reacting anti-*P. falciparum* E1a antibodies (Fig. 1B). The E1a subunit was detected as a ∼45 kDa and a ∼90 kDa band in Western blots of wild type parasites. The 90 kDa band likely corresponds to the E1a/E1b heterodimer, as the intensity of this band was severely diminished under strong denaturating conditions (Fig. 1B). Neither band was detected in the knockout.

The Tg*e1a_ko* formed smaller plaques in a human foreskin fibroblast (HFF) lytic plaque assay compared to the parental RH strain (Fig. 1C) indicating a reduced ability to infect and/or grow in host cells. Further phenotypic analyses revealed that neither Tg*e1a_ko* tachyzoite invasion nor egress from infected host cells were affected (data not shown) and that the reduction in fitness was due to significantly reduced intracellular growth compared to RH parasites, as monitored by the reduced number of parasites per vacuole established by Tg*e1a_ko* after 24 h (Fig. 1D). This phenotype was exacerbated when infected HFF were cultivated in the absence of glucose in a reversible fashion, while removal of glutamine did not aggravate this defect (Fig. 1D).

To validate that the phenotypes observed in Tg*e1a_ko* are only due to the loss of the E1a subunit of BCKDH, we targeted a second copy of the E1a subunit where the N-terminal mitochondrion targeting signal was replaced by the mitochondrial transit signal of TgSOD3 (SOD3mycBCKDH-E1a, Fig. 1E) in Tg*e1a_ko* parasites. In addition, we attempted to complement Tg*e1a_ko* parasites by targeting the product of a second copy of the TgPDH-E1a subunit to the mitochondrion via replacement of its bipartite targeting signal with the mitochondrial transit signal of TgSOD3 (SOD3mycPDH-E1a, Fig. 1E). TgPDH-E1a and TgBCKDH-E1a show significant similarity by sequence alignment (∼25%) and the catalytic residues are clearly conserved between the two subunits (Fig. S3A). The mitochondrial SOD3mycPDH-E1a was unable to rescue the intracellular growth defect of Tg*e1a_ko* parasites while complementation with SOD3mycBCKDH-E1a restored the growth of Tg*e1a_ko* (Fig. 1F). This highlights a lack of permissiveness to interchange subunits between the different α-ketoacid dehydrogenase complexes but moreover confirmed that the phenotypes observed with Tg*e1a_ko* are solely due to the absence of BCKDH activity.

To further examine whether BCKDH is required for virulence, mice were injected intraperitoneally with ∼15 RH or Tg*e1a_ko* tachyzoites. The inoculation of virulent RH parasites resulted in acute toxoplasmosis in all mice after 8 days leading to their culling. In contrast, 3 out of 5 mice infected with Tg*e1a_ko* parasites remained alive after 21 days (Fig. 1G). The surviving mice had seroconverted and were resistant to subsequent challenge with ∼1,000 RH parasites (Fig. 1G). Taken together, these results establish that the BCKDH complex is implicated in glucose catabolism and is important for both parasite fitness *in vitro* and virulence *in vivo*.

### BCKDH is required for catabolism of pyruvate in the TCA cycle of *T. gondii*

**Figure 2:**
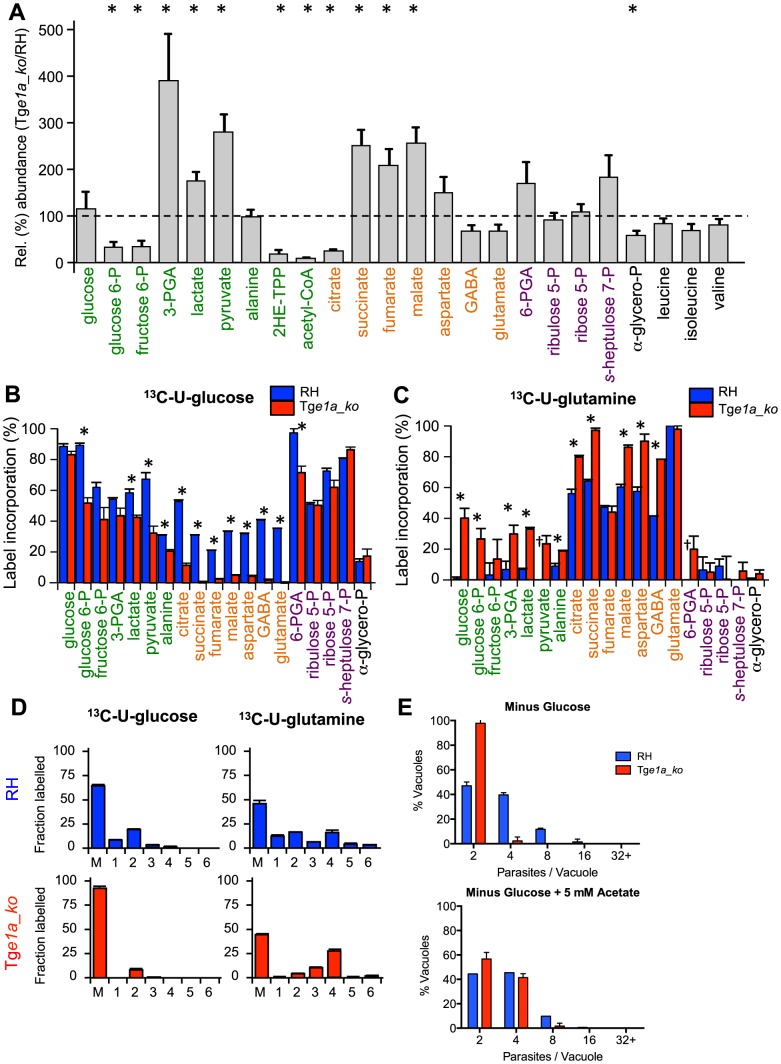
BCKDH is required for conversion of pyruvate to acetyl-CoA and catabolism of glucose in the mitochondrion. Freshly egressed RH and Tg*e1a_ko* tachyzoites were labelled with ^13^C-U-glucose or ^13^C-U-glutamine for 4 h. Abundance and label incorporation were assessed by GC-MS and LC-MS. (A) Relative (%) abundance of selected metabolites in the Tg*E1a_ko* mutant parasites. Bars represent abundance of metabolites in Tg*e1a_ko* cells compared with a parental (RH) control. The dashed line refers the abundance of the metabolite in the parental control (‘100%’). 2HE-TPP refers to 2-hydroxyethyl-thiamine pyrophosphate, the stable intermediate specifically generated by pyruvate dehydrogenase activity. 2HE-TPP and acetyl-CoA were measured by LC-MS while other metabolites were measured by GC-MS (B) ^13^C-glucose and (C) ^13^C-glutamine incorporation into central carbon metabolites in RH (blue) and Tg*e1a_ko* (red) tachyzoites, where label incorporation is the fraction of molecules of that metabolite containing one or more ^13^C carbons (after correction for natural abundance). In A, B and C metabolites are colour-coded by metabolic pathway; central carbon metabolism, green; TCA cycle and associated amino acids, orange; PPP, purple; other, black. Error bars represent standard deviation (n = 3–6). Significance as determined by t-test is shown (corrected for multiple comparisons using the Holm-Sidak method), with significant (p-values of 0.05) differences indicated by an asterisk. † indicates metabolite not detected. (D) Mass isotopologue abundances of citrate generated in ^13^C-glucose and ^13^C-glutamine-fed RH and Tg*e1a_ko* tachyzoites. ‘M’ indicates the monoisotopic mass containing no ^13^C atoms. Error bars indicate standard deviation (n = 3). (E) Intracellular growth of RH (blue) and Tg*e1a_ko* (red) was assessed after 24 h in medium depleted of glucose complemented or not with 5 mM exogenous acetate. Data are represented as means ± SD from three independent biological replicates.

To investigate the underlying basis of the intracellular growth defect in the BCKDH mutant, *T. gondii* RH and Tg*e1a_ko* tachyzoites were cultivated in HFF and metabolite levels in egressed tachyzoites determined by both GC-MS and LC-MS (Fig. 2A). Significant differences were observed in the levels of several glycolytic and early TCA cycle intermediates in Tg*e1a_ko* tachyzoites, compared to the RH control strain. This included a 4- to 10-fold decrease in 2-hydroxyethyl-TPP (the intermediate in synthesis of acetyl-CoA from pyruvate), acetyl-CoA, and citrate, and a 2- to 4-fold increase in 3-phosphoglycerate (3-PGA), pyruvate and lactate (Fig. 2A). These data are consistent with a defect in the conversion of pyruvate to acetyl-CoA and citrate as well as an increased flux to lactate production.

To confirm that Tg*e1a_ko* parasites have a defect in acetyl-CoA synthesis, freshly egressed RH and Tg*e1a_ko* tachyzoites were metabolically labelled with ^13^C-U-glucose. The intermediates in glycolysis, the pentose phosphate pathway (PPP) and the TCA cycle were strongly labelled in RH parasites (Fig. 2B). Citrate isotopomers were generated containing +2, +3 and +4 ^13^C carbons, indicative of entry of both ^13^C_2_-acetyl-CoA and ^13^C_3_-oxaloacetate derived from pyruvate into the TCA cycle of RH parasites (Fig. 2D). In contrast, the labelling of acetyl-CoA and TCA cycle intermediates, including citrate and the C4 dicarboxylic acids, were dramatically reduced in Tg*e1a_ko* (Fig. S2A, 2B and 2D), suggesting that loss of BCKDH is associated with a block in entry of glucose-derived pyruvate into the TCA cycle. This was supported by complementary labelling with ^13^C-U-glutamine, which revealed equivalent or elevated enrichment of label in all TCA cycle intermediates in Tg*e1a_ko* compared to RH parasites (Fig. 2C). The predominant citrate isotopologue generated in ^13^C-glutamine-fed Tg*e1a_ko* had +4 ^13^C atoms (Fig. 2D) indicating that glutamine-derived ^13^C_4_-oxaloacetate combines with a residual source of unlabelled acetyl-CoA to allow citrate synthesis. This could reflect low level capacity of the α-ketoglutarate dehydrogenase to convert pyruvate to acetyl-CoA, or more likely, the conversion of mitochondrial-produced ^13^C_4_-oxaloacetate to citrate in the cytosol via the ATP-citrate lyase or the second putative citrate lyase (TGME49_203110, www.toxodb.org) present in the genome of *T. gondii*. Interestingly, significant labelling of glycolytic intermediates and hexose-phosphate was detected in ^13^C-glutamine-fed Tg*e1a_ko* tachyzoites, which was absent in wild type parasites (RH) (Fig. 2C). Collectively, these findings show that the BCKDH is required for the conversion of pyruvate to mitochondrial acetyl-CoA and operation of a cyclical TCA cycle. In the absence of BCKDH, the continued production of C4 dicarboxylic acids derived from glutamine by the oxidative TCA cycle leads to increased gluconeogenic fluxes despite the fact that these parasites continue to utilize glucose and have a high glycolytic flux.

To investigate whether BCKDH is also required for catabolism of branched chain amino acids (BCAA), RH and Tg*e1a_ko* were labelled with ^13^C-U-leucine, ^13^C-U-isoleucine and ^13^C-U-valine. An untargeted metabolome-wide isotope analysis detected no significant ^13^C-enrichment in TCA cycle intermediates, despite detecting efficient uptake of branched chain amino acids and conversion to the respective ^13^C-labeled branched chain keto acids (data not shown). No significant differences in the steady state levels of leucine, isoleucine or valine were detected between the parental and knock out strains (Fig. 2A). Together these data strongly suggest that mitochondrial acetyl-CoA is not derived from BCAAs under normal growth conditions (Fig. S2B).

To determine whether the role of BCKDH in acetyl-CoA production could be by-passed by addition of exogenous acetate, fibroblasts infected with Tg*e1a_ko* were cultivated in media with or without acetate. Supplementation of the medium with acetate led to a partial but significant rescue of the severe growth defect observed in the absence of glucose (Fig. 2E). This result is consistent with BCKDH having a role in acetyl-CoA production and suggests some redundancy in the functions of BCKDH and acetyl-CoA synthetase in generating mitochondrial and/or cytoplasmic pools of acetyl-CoA.

### BCKDH is a PDH-like enzyme

**Figure 3:**
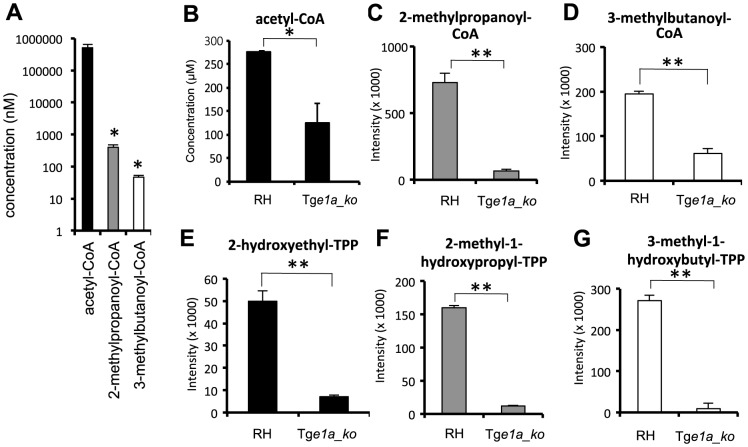
BCKDH possesses PDH activity. (A) *In vitro* enzyme activity indicates a low level of classical branched chain keto-acid dehydrogenase activity and extensive pyruvate dehydrogenase activity in RH *T. gondii* cell lysates. Concentrations (mean ± SD) of acetyl-CoA (black), 2-methylpropanoyl-CoA (grey), and 3-methylbutanoyl-CoA (white columns), were measured following incubation of RH lysates with 2 mM pyruvate (black), 3-methyl-2-oxobutanoate (grey) or 4-methyl-2-oxopentanoate (white columns), respectively. (B) concentration of acetyl-CoA following *in vitro* incubation of RH or Tg*e1a_ko* extracts with 0.5 mM pyruvate (C–D) Relative abundance of acyl-CoA products following *in vitro* incubation of RH or Tg*e1a_ko* extracts with 0.5 mM (C) 3-methyl-2-oxobutanoate or (D) 4-methyl-2-oxopentanoate. (E–G) Relative abundance of hydroxyalkyl-TPP intermediates following incubation of RH or Tg*e1a_ko* lysates with 0.5 mM (E) pyruvate, (F) 3-methyl-2-oxobutanoate or (G) 4-methyl-2-oxopentanoate. Metabolite intensity (y-axis) is measured by LC-MS peak area (mean ± SD; n = 2). Significance as determined by t-test is shown, with p-values of <0.05 and <0.01 indicated by an asterisk and double asterisk, respectively.

*In vitro* enzyme assays were performed to investigate the substrate selectivity of *T. gondii* BCKDH. As attempts to express an active, recombinant BCKDH complex were unsuccessful, enzyme assays were performed on whole cell lysates from RH and Tg*e1a_ko* parasites. PDH activity was detected when cell lysates were incubated with 0.5 mM pyruvate in the presence of cofactors, with over two-fold higher concentration of acetyl-CoA production observed in RH (276 µM) than Tg*e1a_ko* (124 µM) extracts, confirming a role of BCKDH in acetyl-CoA production (Fig. 3B) (p<0.05). The significant level of background PDH-like activity observed in Tg*e1a_ko* extracts is likely mediated by the apicoplast PDH or mitochondrial α-KDH complexes. Minimal production of the branched chain acyl-CoAs, 3-methylpropanoyl-CoA (128 nM) and 3-methylbutanoyl-CoA (below limit of quantitation; LOQ = 5 nM), was detected following incubations with their respective substrates, 4-methyl-2-oxopentanoate and 3-methyl-2-oxobutanoate. Branched chain acyl-CoA formation was significantly lower in Tg*e1a_ko* compared to RH extracts (Fig. 3C–D), suggesting BCKDH does indeed possess classical BCKDH-like activity. However, accurate quantification of acyl-CoA products from assays with higher substrate concentrations (2 mM) confirmed that the BCKDH-like activity was 1000- to 10,000-fold lower than the PDH-like activity (Fig. 3A), suggesting that this enzyme functions primarily as a PDH *in vivo*. Interestingly, the hydroxyalkyl-TPP intermediates for all three substrates were detected in a BCKDH-dependent manner (Fig. 3E–G).

### BCKDH activity is required for correct intraerythrocytic development of *P. berghei*

**Figure 4:**
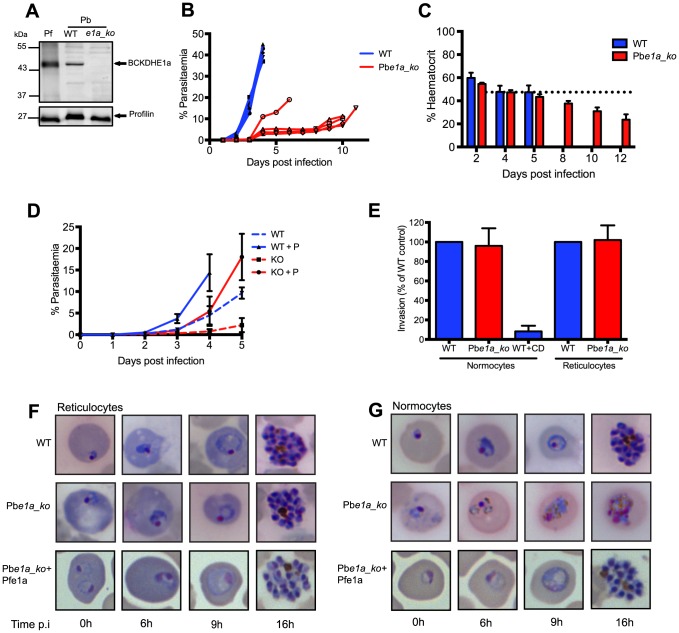
BCKDH is required for growth of *Plasmodium berghei* in mature RBCs. (A) Total lysates from a mixed population of parasitic stages for *P. falciparum* 3D7 (Pf), Pb wild type (WT) and Pb*e1a_ko* were analysed by Western blot. Expression of BCKDH-E1a (shown by the arrow) was assessed using cross-reacting anti-PfBCKDH-E1a antibodies. Profilin was used as loading control. (B) Parasitaemia was followed daily in mice infected with WT (blue line) or Pb*e1a_ko* (red line). Each line corresponds to the parasitaemia of one mouse. 5 mice were infected per condition. (C) Haematocrit was followed over the course of infection in mice infected with WT (blue) or Pb*e1a_ko* (red). Corresponding parasitaemia levels of this experiment are shown in Fig. S3B. The dotted line represents the mean haematocrit level of uninfected control mice followed throughout the experiment. Data show mean ± SD from 4 mice per condition. (D) Parasitaemia was followed daily in mice pre-treated (full lines) or not (dotted lines) with phenylhydrazine to induce reticulocytosis 3 days prior infection with parasites. Mice were infected with 5×10^6^ WT (blue line) or Pb*e1a_ko* (red line) parasites. 5 mice were infected per condition and lines represent mean parasitaemia ± SD. (E) Invasion of Pb*e1a_ko* (red) compared to WT (blue). Vybrant Green-labeled purified schizonts were incubated with DDAO-SE-labeled purified normocytes or reticulocytes, and free merozoites were allowed to invade. Parasitaemia in the DDAO-SE-labeled target population was determined by flow cytometry. Invasion efficiency was determined as a percentage of the WT control parasites. Cytochalasin D (CD)-treated schizonts were used as a negative control. Data are represented as mean ± SD of three independent biological replicates. (F) Giemsa-stained blood smears showing normal development of WT, Pb*e1a_ko* and complemented Pb*e1a_ko*+Pfe1a parasites to the schizont stage in purified reticulocytes. Parasites were cultured *in vitro* for the times indicated. (G) Giemsa-stained blood smears showing development of the different strains within purified normocytes. WT parasites mature normally from ring to schizont stage while Pb*e1a_ko* degenerate rapidly. Complemented Pb*e1a_ko*+Pfe1a restored the ability of Pb*e1a_ko* to develop within normocytes. Parasites were cultured *in vitro* for the times indicated.

To determine whether BCKDH has a similar role in malaria parasites, a *P. berghei* mutant lacking the BCKDH E1a subunit was generated by double homologous recombination (Pb*e1a_ko*) (Fig. S4A). Several independent positive transgenic pools were obtained after drug cycling. However, their slow growth hampered the cloning of the mutants by limiting dilution in wild type immunocompetent CD1 mice. Since the parasites seemed to exhibit a severe fitness defect that might lead to clearance of the infection by the mouse immune system, we switched to immunodeficient RAG-1 -/- mice for cloning purposes [37]. Loss of expression of Pb*e1a* in the clonal Pb*e1a_ko* line from these mice, was demonstrated by genomic PCR and Western blot analysis (Fig. S4D and 4A). Strikingly, mice infected with 15×10^6^ Pb*e1a_ko* parasites had constant low parasitaemias (5–15% over 10 days), while infection with WT parasites led to an exponential rise in parasitaemia (up to 45% after 4 days), leading to culling due to illness (Fig. 4B). Despite having much lower parasitaemias, mice infected with Pb*e1a_ko* parasites developed symptoms of severe anaemia and were culled 10–12 days post-infection (Fig. 4B). The haematocrit level between the mice infected with WT or Pb*e1a_ko* was comparable during the first five days of infection (Fig. 4C). Haematocrit levels continuously decreased over the subsequent 7 days in the Pb*e1a_ko*-infected mice (Fig. 4C), consistent with the observed anaemia in these animals.

To understand why the haematocrit level decreased despite relatively low parasitaemia, parasite distribution was further investigated in the different red blood cell types. In wild type parasite infected mice, parasites could be found both within reticulocytes as well as in normocytes. In contrast, the majority of Pb*e1a_ko* parasites were present within reticulocytes throughout the course of infection. To assess the importance of this apparent cell tropism, we induced reticulocytosis in mice with phenylhydrazine prior to infection. In mice infected with wild type, parasitaemia increased as expected and phenylhydrazine pre-treatment slightly accentuated the growth of the parasites. Pre-treatment of mice with phenylhydrazine rescued significantly the growth defect observed with Pb*e1a_ko* (although not to wild type levels as reticulocytes maturate into normocytes over the course of infection) while in mice not pre-treated the parasitaemia levels remained low throughout the 5 days of infection supporting the observation that Pb*e1a_ko* seemed to preferentially infect reticulocytes (Fig. 4D). This differential distribution is not explained by an invasion defect of the Pb*e1a_ko* parasites for normocytes as shown in (Fig. 4E). Indeed, we observed no difference in invasion efficiency between WT and Pb*e1a_ko* parasites when using purified normocytes and reticulocytes as target host cells.

*In vitro* maturation was examined following *in vitro* invasion of purified normocytes or reticulocytes. This selective distribution appears to be due to rapid loss of viability of Pb*e1a_ko* in the normocytes. Specifically, WT parasites developed to the schizont stage in both reticulocytes and normocytes (Fig. 4F and 4G, respectively), whereas Pb*e1a_ko* parasites developed normally in reticulocytes (Fig. 4F), but rapidly degenerated within normocytes (Fig. 4G). Taken together, these findings demonstrate that a functional BCKDH complex is required for the development of *P. berghei* in mature erythrocytes. The abortive infections of normocytes are most likely eliminated from the circulation by the spleen and liver, resulting in the lower parasitaemia and protracted course of infection of the mutant.

To confirm that the observed attenuation phenotype is solely attributable to the deletion of the *PbBCKDH-E1a* gene, Pb*e1a_ko* parasites were complemented with a copy of the *P. falciparum BCKDH*-*E1a* gene, PfE1a (Fig. S4C). Pb*e1a_ko*+PfE1a parasites were obtained after several passages in mice with intermittent drug selection. Integration of the complementation plasmid and PfE1a protein expression were confirmed by genomic PCR (Fig. S4D) and Western blot analyses (Fig. S4E). Complementation with PfE1a restored the ability of these parasites to complete their development in mature erythrocytes (Fig. 4F and 4G) and their growth rate *in vivo* was comparable to the parental WT strain (Fig. S4F). These results also indicate that the E1a subunit from *P. falciparum* can assemble with the *P. berghei* subunits to form a functional BCKDH multi-enzyme complex.

### BCKDH acts as a PDH-like complex in the mitochondrion of *P. berghei*

**Figure 5:**
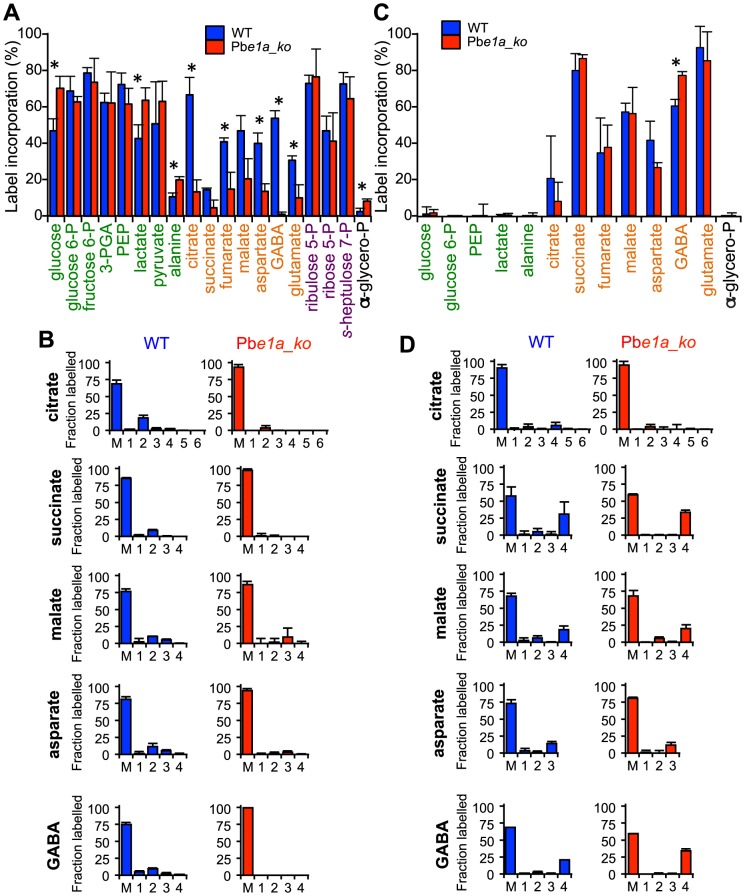
*P. berghei* parasites lacking the BCKDH-E1a subunit exhibit a perturbed TCA cycle. Cultures containing ring/early-trophozoite WT and Pb*e1a_ko P. berghei*-infected RBCs were allowed to mature to schizonts and labelled with ^13^C-U-glucose or ^13^C-U-glutamine for 5 hr. Label incorporation was assessed by GC-MS. (A) Total ^13^C-glucose-derived label incorporation into central carbon metabolism metabolites in WT (blue) and Pb*e1a_ko* (red) *P. berghei*-infected RBCs, where label incorporation is the fraction of molecules of that metabolite containing one or more ^13^C carbons (after correction for natural abundance). Metabolites are colour-coded by metabolic pathway; central carbon metabolism, green; TCA cycle and associated amino acids, orange; PPP, purple; other, black. Error bars represent standard deviation (N = 4). Significance as determined by t-test is shown (corrected for multiple comparisons using the Holm-Sidak method), with significant (p-value<0.05) differences indicated by an asterisk. † indicates metabolite not detected. (B) Mass isotopologue distributions of the TCA intermediates shown in Panel A. The x-axis indicates the number of ^13^C-atoms in each metabolite (‘M’ indicates the monoisotopic mass containing no ^13^C atoms). The y-axis indicates fractional abundance of each isotopologue when labelled with ^13^C-U-glucose (present in the culture medium at ∼50%). Error bars indicate standard deviation (N = 4). (C, D) As for A and B, but after labelling with ^13^C-U-glutamine (present in the culture medium at ∼98%).

To determine whether the BCKDH complex fulfils the function of a mitochondrial PDH in the *P. berghei* asexual blood stages, ring/early trophozoite stages of WT and Pb*e1a_ko* parasite-infected RBCs (iRBCs) were matured to schizonts *in vitro* and labelled with ^13^C-U-glucose or ^13^C-U-glutamine over the final 5 h of maturation. Schizont-iRBCs were purified and the labelling of intracellular metabolite pools determined by GC-MS. Intermediates in glycolysis, the PPP and TCA cycle were highly enriched when RBCs infected with WT parasites were labelled with ^13^C-glucose (Fig. 5A and S5A) while in uninfected RBCs, ^13^C-labelling from glucose was only detected in the glycolysis and PPP metabolites (Fig. S5A). After 5 h labelling, the major isotopologues of citrate in WT iRBCs contained two ^13^C-carbons, reflecting the incorporation of ^13^C_2_-acetyl-CoA into citrate after one round of the TCA cycle (Fig. 5B). While a similar labelling of glycolytic and PPP intermediates was observed in Pb*e1a_ko*-iRBC, incorporation into TCA intermediates and associated amino acids was greatly diminished in the Pb*e1a_ko* compared to WT iRBCs (Fig. 5A, 5B and S5A). It is notable that +3 isotopologues of malate and aspartate (a proxy of oxaloacetate) were still detected in ^13^C-glucose-fed parasites, reflecting continued conversion of pyruvate to malate (via oxaloacetate) by the action of PEPC (Fig. 5B), as recently observed [23]. These findings demonstrate that BCKDH is required for the mitochondrial catabolism of glucose by conversion of pyruvate to acetyl-CoA in *P. berghei* blood stage parasites.

Importantly, both wild type and Pb*e1a_ko*-infected RBC catabolized ^13^C-U-glutamine in a canonical TCA cycle (Fig. 5C and 5D), as has recently been shown to occur in *P. falciparum* [21]–[23]. However, in contrast to the situation in *T. gondii*, no evidence for increased gluconeogenesis in the Pb*e1a_ko*-infected RBC was observed, consistent with the absence of key gluconeogenic enzymes in these parasites [10].

As anticipated, label derived from ^13^C-U-leucine was not incorporated into TCA cycle intermediates in either WT, Pb*e1a_ko*-infected RBC, or uninfected RBCs (Fig. S5B), indicating that PbBCKDH does not catabolise α-ketoacids generated from BCAA and consistent with the absence of BCAA-specific aminotransferase (BCAT) in the malaria parasite genomes (Table S1).

**Figure 6:**
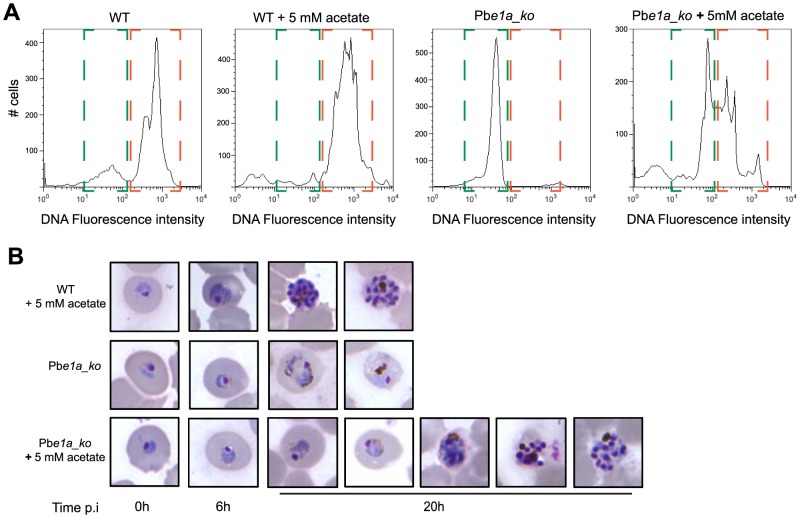
Acetate complementation of *P.berghei* BCKDH null mutants. (A) Following *in vitro* invasion of normocytes with WT or Pb*e1a_ko*, parasites were allowed to mature *in vitro* for 20 h in media supplemented or not with 5 mM acetate. Replication of DNA content was taken as measure of parasite maturation. DNA was labelled using Vybrant DyeCycle Ruby Stain and fluorescence intensity was measured by flow cytometry. Highlighted in green is the ring/parasites degenerated early in their development fraction while trophozoite/schizont stage iRBCs are highlighted in orange. The results of two other biological replicate can be found in Fig. S6. (B) Giemsa-stained blood smears showing development of the different strains within normocytes in medium complemented or not with 5 mM acetate. WT mature from ring to schizonts within normocytes in presence of 5 mM acetate. Pb*e1a_ko* degenerate rapidly within normocytes in normal culture conditions while complementation with 5 mM acetate rescues partially their viability and maturation. Figure shows the various stages of Pb*e1a_ko* maturation that can be observed over the *in vitro* culture period. Parasites were cultured *in vitro* for the times indicated.

To assess whether differences in the acetyl-CoA levels of Pb*e1a_ko*, via conversion of acetate to acetyl-CoA from the acetyl-CoA synthetase, could be responsible for the reticulocyte tropism that is observed, we followed *in vitro* maturation of wild type and Pb*e1a_ko* parasites within normocytes in presence or not of exogenous acetate. Pb*e1a_ko* parasite growth in normocytes was partially restored by supplementation of the medium with acetate (Fig. 6A–B and S6A), indicating that the presence of acetate in reticulocytes could be one of the factors contributing to the ability of Pb*e1a_ko* parasites to survive and develop within this cell type but not within normocytes.

### BCKDH has roles in *P. berghei* sexual development and oocyst maturation

**Figure 7:**
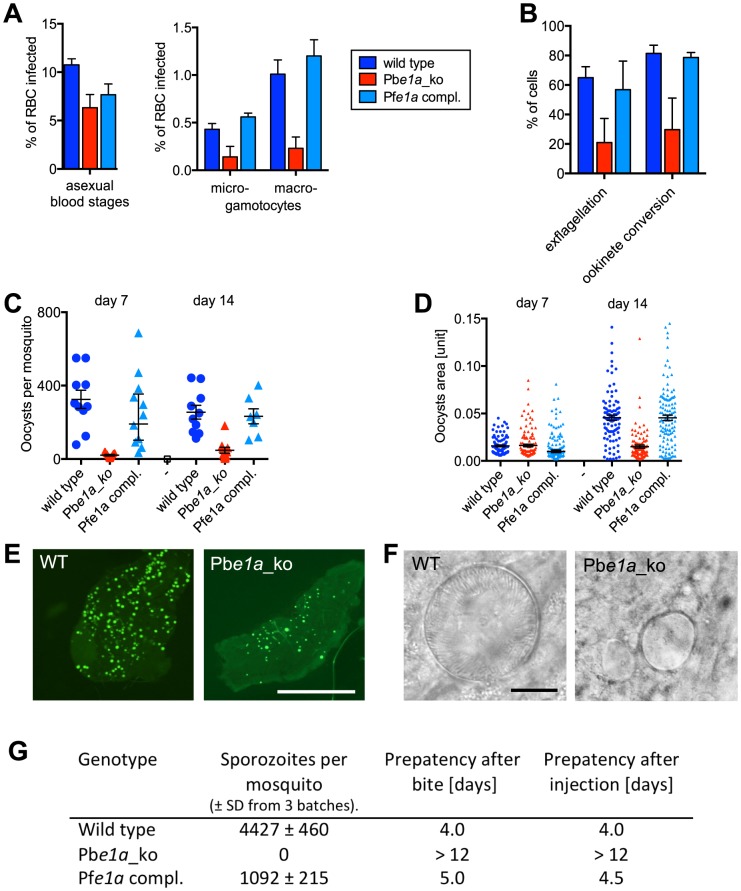
Role of BCKDH during sexual development and in mosquito stages of *P. berghei*. (A) Asexual parasitaemia and male (micro-) and female (macro-) gametocytaemia in the peripheral blood of mice 3 days post infection with 1×10^7^ parasites i. p. Error bars show standard deviations from 3 mice. (B) Developmental capacity of gametocytes *in vitro* measured from the same infections shown in panel A. The relative ability of microgametocytes to release microgametes was assessed by counting exflagellation centres in a haemocytometer 15 min after addition to activating medium. The ability of activated macrogametocytes to become fertilised and convert to ookinetes was assessed by quantifying round and ookinete-shaped parasites following life staining of the surface marker P28. Colour code as in panel A; error bars show standard deviations. (C) Oocyst numbers on the midguts of individual *A. stephensi* mosquitoes on different days after feeding on three infected mice per mutant. Geometric means and 95% confidence intervals are also shown. (D) Sizes of individual oocysts from infected midguts at different days after infection. Black lines show geometric means and 95% confidence intervals. (E) Fluorescence micrographs of representative *A. stephensi* midguts dissected 14 days after feeding on wild type and mutant parasites expressing GFP. Scale bar = 0.5 mm. (F) Phase contrast images of representative oocysts. Scale bar = 10 µm. (G) Sporozoite numbers per mosquito as determined from 3 batches of 10 dissected salivary glands. Transmission from mosquito to mice was examined by measuring the prepatent period in 2 mice per group after bites from ∼20 mosquitoes or intraperitoneal injection of homogenates from 10 pairs of salivary glands. Data shown in all panels are representative of two independent experiments each performed with three infected mice per parasite line.

We next assessed the effect of deleting the *BCKDH-E1a* gene on sexual development and mosquito transmission of *P. berghei*. To minimise a potential indirect effect of the attenuated growth of the Pb*e1a_ko* mutant on gametocyte numbers, mice were injected with phenylhydrazine two days before infection, inducing mild anaemia and increased reticulocytosis. As a result, a similar parasitaemia was obtained for all strains on day 3 post-infection (Fig. 7A). The numbers of morphologically mature female (macro-) and male (micro-) gametocytes were lower in Pb*e1a_ko* parasites than in either wild type or PfE1a complemented clones (Fig. 7A). The small number of microgametocytes in the Pb*e1a_ko* clone had a considerably reduced capacity to differentiate (exflagellate) into microgametes when stimulated by xanthurenic acid *in vitro*. Possibly as a result, the ability of macrogametocytes to convert into ookinetes upon activation *in vitro* was also reduced (Fig. 7B). Absence of BCKDH thus reduced both the numbers of morphologically mature gametocytes and their ability to develop *in vitro*.

To assess the role of BCKDH in transmission, *Anopheles stephensi* mosquitoes were allowed to feed on infected mice. Consistent with the *in vitro* data, the Pb*e1a*_ko mutant gave rise to a considerably reduced number of oocysts per infected mosquito midgut, an effect that was fully reversed by complementation with the PfE1a gene (Fig. 7C). Importantly, after day 7 of infection, Pb*e1a*_*ko* showed a marked inability to increase in size (Fig. 7D and 7E), and the mutant cysts invariably failed to undergo sporogony (Fig. 7F). Pb*e1a_ko*-infected mosquitoes contained no sporozoites in their salivary glands on day 21 post infection, neither were they able to re-infect mice, either by bite or intravenous injection of disrupted salivary glands (Fig. 7G). Taken together, these data demonstrate that in addition to its role for blood stage development in normocytes, BCKDH is necessary for normal gametocyte production and fitness, and that BCKDH is essential for oocysts to mature and undergo sporogony.

## Discussion

Using a combination of genetic and metabolomic approaches, we show that the BCKDH complex not only substitutes for the loss of mitochondrial PDH in *Apicomplexa*, but is also required for normal growth and virulence of *T. gondii* and *P. berghei*. While *T. gondii* and *Plasmodium* spp. were thought to depend on glycolysis for energy, recent studies have highlighted the potential importance of oxidative phosphorylation and mitochondrial metabolism [27], [28], [30], [38]. Indeed ^13^C-glucose and ^13^C-glutamine labelling studies have recently confirmed the operation of a canonical TCA cycle in both *T. gondii* and *P. falciparum* [20], [21]. A critical question raised by these studies concerns the identity of the enzyme(s) that feed carbon skeletons into the TCA cycle. In most organisms, the operation of the TCA cycle is dependent on production acetyl-CoA from pyruvate, via the catalytic activity of a mitochondrial PDH complex. *Apicomplexa* have a canonical PDH, but this complex is targeted to the apicoplast, suggesting that other enzymes fulfil the function of the PDH in the mitochondrion [13]–[15]. Recent studies have raised the possibility that mitochondrial acetyl-CoA could be generated from glycolytic pyruvate via one of the other TPP-dependent mitochondrial dehydrogenases [22], [33]. However, direct evidence for involvement of either BCKDH or α-KDH, or another uncharacterized dehydrogenase has not been obtained.

Here, we have generated *T. gondii* and *P. berghei* BCKDH null mutants by reverse genetics and demonstrated loss of mitochondrial glucose metabolism. Deletion of the *BCKDH-E1a* gene from *T. gondii* and *P. berghei* was non-lethal, but in each case led to a marked reduction in growth rate and impacted on parasite virulence in mice. Metabolite profiling coupled with ^13^C-U-glucose labelling established that BCKDH catalyses the conversion of mitochondrial pyruvate to ^13^C_2_-acetyl-CoA and fuels a canonical TCA cycle both in *T. gondii* and *P. berghei*. Specifically, deletion of *BCKDH-E1a* was associated with loss of labelling of ^13^C_2_-acetyl-CoA and +2 ^13^C-isotopologues of other TCA cycle intermediates. In *T. gondii*, loss of the *BCKDH-E1a* gene also led to significant decreases in 2-hydroxyethyl-TPP, the intermediate in acetyl-CoA production from pyruvate, as well as acetyl-CoA and citrate. Conversely, pyruvate levels increased in a manner consistent with this substrate no longer being consumed by the enzyme. *In vitro* analysis of α-ketoacid dehydrogenase activity in *T. gondii* extracts demonstrated some affinity for both pyruvate and branched-chain keto-acids, but with a high selectivity for the conversion of pyruvate to acetyl-CoA compared to the minimal conversion of branched chain keto-acids to their respective branched chain acyl-CoA products. Taken together, our data demonstrate that the apicomplexan BCKDH complex has been repurposed to function as a PDH and allow the further oxidation of pyruvate in a canonical TCA cycle.

Sequence alignment analysis of the *T. gondii* and *P. berghei* E1a with other BCKDH-E1a (Fig. S3B and S3C) and E1b subunits failed to ascertain whether active site residue substitutions can account for substrate versatility [39], [40] and further structural characterization will be required to refine our understanding of the substrate specificity of this class of enzyme. However, these results account for the retention of BCKDH genes in *Plasmodium* spp., despite the loss of other genes involved in BCAA degradation. They are also consistent with the presence of the two genes coding for the subunits of the mitochondrial pyruvate carrier recently described in yeast [41], [42] (Table S1). Interestingly, more distantly related members of the *Alveolata*, such as *dinoflagellates* also lack a conventional mitochondrial PDH complex, but retain a BCKDH [17], suggesting that the repurposing of BCKDH evolved early in the evolution of this group and is likely to be conserved in all members of this group that contain a functional TCA cycle.

**Figure 8:**
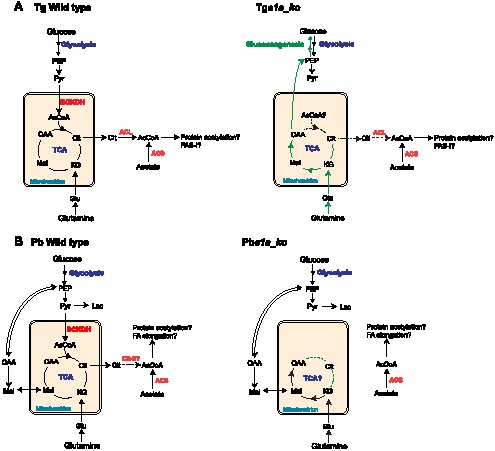
Proposed metabolic pathways, compartmentalisation and metabolic remodelling in *T. gondii* and *P. berghei*. (A) Schematic representation of the metabolism in *T. gondii* WT (left panel) and Tg*e1a_ko* (right panel) incorporating data from this study. (B) Scheme of the metabolism in *P. berghei* WT (left panel) and Pb*e1a_ko* (right panel). For both (A) and (B), the remodelling of the parasites metabolism upon ablation of BCKDH activity is highlighted in green. Dotted lines represent drops and disruption in the corresponding reactions. Abbreviations: AcCoA, acetyl-CoA; α-KG, α-ketoglutarate; Cit, citrate; Glc, glucose; Glu, glutamate; Gln, glutamine; Lac, lactate; Mal, malate; OAA, oxaloacetic acid; PEP, phosphoenolpyruvate; Pyr, pyruvate; Suc, succinate. Enzymes in red: BCKDH, branched chain keto acid dehydrogenase; ACL, ATP-citrate lyase; ACS, Acetyl-CoA synthetase; CS-II, second isoform of citrate synthetase.

Intriguingly, the disruption of the BCKDH enzyme complex was associated with significant remodelling in the central carbon metabolism in *T. gondii* (Fig. 8). Metabolic profiling of Tg*e1a_ko* parasites revealed that despite a decrease in citrate, other TCA cycle metabolites including the C4-dicarboxylic acids succinate, fumarate and malate were all unaltered or even increased in abundance, indicating that an alternative carbon source enters the TCA cycle below α-ketoglutarate. *T. gondii* tachyzoites co-utilize glutamine in the presence of glucose and appear to use this amino acid as an alternative substrate in the absence of glucose [43]. Unexpectedly, we found that carbon skeletons derived from ^13^C-glutamine were channelled into the gluconeogenic pathway in Tg*e1a_ko* parasites under glucose-replete conditions (Fig. 2C and 8). This metabolic perturbation is not due to interruption of the early steps in the TCA cycle or increased glutaminolysis, since increased gluconeogenic flux is not observed in parasites treated with sodium fluoroacetate, an inhibitor of the TCA cycle enzyme aconitase [20]. Increased gluconeogenesis in Tg*e1a_ko* might result from allosteric activation of key enzymes, as a result of the accumulation of pyruvate or other metabolites. Alternatively, the decreased production of acetyl-CoA in the mutant could lead to global changes in the acetylation state of multiple enzymes in these pathways [44]–[46]. The gluconeogenic enzyme, phosphenolpyruvate carboxykinase (PEPCK) is activated by deacetylation [47], and this enzyme has been shown to be acetylated in *T. gondii* tachyzoites, as is cytosolic glyceraldehyde-3-phosphate dehydrogenase (GAPDH) [48]. Bacterial acetylated GAPDH has been reported to drive the forward reaction during glycolysis, while it is more active in catalysing the reverse reaction used in gluconeogenesis when deacetylated [45]. A global decrease in protein acetylation as a consequence of blocked acetyl-CoA synthesis could therefore underlie the inappropriate activation of gluconeogenesis in Tg*e1a_ko* (Fig. 8).

The phenotypic analyses of the BCKDH null mutant revealed an important impact on the physiology of the blood stages of the rodent malaria parasite. In contrast to other *P. berghei* mutants with defects in central carbon metabolism, such as the mitochondrial complex II (succinate-ubiquinone reductase) [28] and the type II NADH:ubiquinone oxidoreductase (NDH2) [27], the Pb*e1a_ko* mutant exhibited an obvious phenotypic defect during asexual development in mature red blood cells. Pb*e1a_ko* parasites readily invaded reticulocytes and normocytes, but failed to develop to schizonts in the latter. While the molecular basis for the selective growth in reticulocytes compared to normocytes is unknown, it is likely that differences in the metabolism of these two host cell types contribute to the observed tropism. In particular, reticulocytes are known to be metabolically more active than normocytes, and may contain essential metabolites that could compensate for the loss of BCKDH function [49], [50]. In support of this conclusion, Pb*e1a_ko* parasite growth in normocytes was partially restored by supplementation of the medium with acetate (Fig. 6 and 8), indicating that the mutant is able to scavenge acetate or other essential nutrients from reticulocytes in order to survive in the absence of BCKDH. Alternatively, intrinsic- and parasite-induced differences in the permeability of the reticulocyte/normocyte plasma membrane [51]–[53] could lead to a differential accumulation of toxic compounds, such as lactate and pyruvate, and lethality in the Pb*e1a_ko* strain. These findings also raise the question of the importance of BCKDH for each of the different human malaria parasite species, given that *P. falciparum* can develop in both immature and mature erythrocytes, whereas *P. vivax* exhibits a strict preference for reticulocytes [54] and establishes a chronic infection in the liver [55].

We have recently shown that flux of pyruvate derived from glucose into the TCA cycle increases markedly as *P. falciparum* asexual RBC stages differentiate to gametocyte stages, implying that the steps catalysed by BCKDH may be regulated in a stage-specific manner [21]. Consistent with an increased role for *P. falciparum* BCKDH in sexual development, the *P. berghei* BCKDH-E1a mutant exhibited a reduction in both numbers and fitness of gametocytes. Both phenotypes were reversed by complementing the mutant with an orthologous gene from *P. falciparum*. While a reduction in gametocyte numbers alone would be difficult to interpret given the different infection courses of wild type and mutant parasites, a reduction in the ability of each microgametocyte to release microgametes and of macrogametes to convert to ookinetes is highly significant. Our phenotype is clearly less severe, but otherwise resembles that described by [28] in *P. berghei* for a mutant in mitochondrial complex II, predicted to have a disrupted TCA cycle downstream of both glucose and glutamine. These data are entirely consistent with a key role for BCKDH in a glucose-fuelled TCA cycle that in *P. berghei* increases in importance during sexual development.

The *P. berghei* BCKDH-E1a is essential for transmission through mosquitoes as it is required for sporogony, a period when the young oocysts grow rapidly in size and differentiate into thousands of sporozoites. Oocyst growth was also blocked in an NDH2-deficient *P. berghei* mutant [27], further highlighting the importance of oxidative phosphorylation during these stages of parasite development. Together, our data suggest that BCKDH plays a key role in the stage-specific changes in flux of glycolytic pyruvate into the TCA cycle in mosquito stages.

This study highlights the metabolic adaptations that have occurred during the evolution of this diverse group of protists and provides new insights into the central carbon metabolism of key pathogenic stages. Since perturbation of mitochondrial metabolism results in significant decrease in parasite fitness in the mammalian host and inhibition of vector transmission, components of the BCKDH complex may be suitable targets for drug development.

## Materials and Methods

### Ethics statement

All animal experiments were approved and performed in accordance with a project licence issued by the UK Home Office and by the Direction générale de la santé, Domaine de l’expérimentation animale (Avenue de Beau-Séjour 24, 1206 Genève) with the authorization Number (1026/3604/2, GE30/13) according to the guidelines set by the cantonal and international guidelines and regulations issued by the Swiss Federal Veterinary Office.

No human samples were used in these experiments. Human foreskin fibroblasts (HFF) were obtained from ATCC.

### Antibodies

The antibodies used in this study were described before as follows: mouse monoclonal anti-myc (9E10), polyclonal rabbit anti-GAP45 [56], rabbit anti-PfProfilin [56]. For the production of specific polyclonal antibodies, PfBCKDH-E1a sequence (PF13_0070, aa 277 to 425) was amplified using primers 2391 and 2392 (Table S2) and cloned into pETHb in frame with 6 histidine residues between the NcoI and SalI restriction sites. The corresponding recombinant protein was expressed in *Escherichia coli* BL21 strain and purified by affinity chromatography on Ni-NTA agarose (Qiagen) according to the manufacturer’s protocol under denaturing conditions. Antibodies against PfBCKDH-E1a were raised in rabbits by Eurogentec S.A. (Seraing, Belgium) according to their standard protocol.

Immunofluorescence assay (IFA) and Western blots analysis with these antibodies were performed as previously described [33].

### *T. gondii* strains and culture

*T. gondii* tachyzoites (RH*ku80_ko* (RH), RH*ku80_ko/bckdhE1a_ko* (Tg*e1a_ko*)) were maintained in HFF using Dulbecco’s Modified Eagle’s Medium (DMEM, GIBCO, Invitrogen) supplemented with 5% foetal calf serum, 2 mM glutamine and 25 µg/ml gentamicin at 37°C and 5% CO_2_.

### Generation *T. gondii* Tg*e1a_ko*

2 kb genomic flanking sequences (FS) of Tg*BCKDH-E1a* (TGME49_239490, www.toxodb.org) ORF were amplified using LATaq polymerase (TaKaRa) and primers 2821 and 2822 for the 5’FS and primers 2823 and 2824 for the 3’FS (Table S2). Amplified regions were then cloned into the *Kpn*I, *Xho*I sites for the 5’FS and *Bam*HI, *Not*I sites for the 3’FS of the pTub5HXGPRT vector. *T. gondii* RH*ku80_ko* tachyzoites were transfected by electroporation as previously described [57] and stable transfectants were selected for by hypoxanthine-xanthine-guanine-phosphoribosyltransferase (HXGPRT) expression in the presence of mycophenolic acid and xanthine as described earlier [58]. Parasites were cloned by limiting dilution in 96 well plates and clones were assessed by genomic PCR and Western blot analysis.

### Phenotypic analyses

Plaque assays: HFF monolayers were infected with parasites and let to develop for 7 days before fixation with PFA/GA and Giemsa staining (Sigma-Aldrich GS500) mounted with Fluoromount G and visualized using ZEISS MIRAX imaging system equipped with a Plan-Apochromat 20×/0.8 objective at the bioimaging facility of the Faculty of Medicine, University of Geneva.

Intracellular growth assays: Prior to infection, the HFF monolayers were washed and pre-incubated for 24 h with medium containing the relevant carbon source and kept in this medium for the rest of the experiment. Complete medium is DMEM 41966 (Gibco, Life Technologies) supplemented with 5% FCS, up to 6 mM glutamine, 25 µg/ml gentamicine. Medium depleted in glutamine is DMEM 11960 supplemented with 25 µg/ml gentamicine (Gibco, Life Technologies) and medium depleted in glucose is DMEM 11966 supplemented with up to 6 mM glutamine and 25 µg/ml gentamicine. HFF were inoculated with parasites and coverslips were fixed 24 h post-infection with 4% PFA and stained by IFA with rabbit anti-TgGAP45, mouse anti-myc 9E10. Number of parasites per vacuole was counted in triplicates for each condition (n = 3). More than 200 vacuoles were counted per replicate.

### *In vivo* virulence assay

On day 0, mice were infected by intraperitoneal injection with either wild type RH or Tg*e1a_ko* parasites (∼15 parasites per mouse). 5 female CD1 mice were infected per group. The health of the mice was monitored daily until they presented severe symptoms of acute toxoplasmosis (bristled hair and complete prostration with incapacity to drink or eat) and were sacrificed on that day in accordance to the Swiss regulations of animal welfare. 21 days post-infection, sera from mice that survived primary infection were assessed for seroconversion by Western blot for the presence of anti-*T. gondii* antibodies. Mice were then challenged with ∼1000 wild-type RH parasites to assess immunization and survival.

### *Plasmodium berghei* culturing

The *P. berghei* ANKA GFP-con clone 2.3.4 was used to generate transgenic parasites and maintained in female CD1 or Theiler's Original outbred mice as described previously [59]. The course of infection was monitored on Giemsa-stained tail blood smears.

### Generation of *P. berghei* transgenic line

Pb*BCKDH-E1a* sequence was retrieved from the online *Plasmodium* genome database, (PBANKA_141110, www.plasmodb.org). To generate the Pb*BCKDH-E1a* knockout (Pb*e1a_ko*), primers 3835 and 2482 were used to amplify a 2 kb region of homology at the 5’end of the Pb*BCKDH-E1a* locus (Table S2). The PCR fragment was cloned between *Kpn*I and *Apa*I restriction sites of the pBS-DHFR vector containing the *T. gondii dhfr* conferring pyrimethamine resistance [60]. The 3’ flanking region 2 kb was amplified using primers 3836 and 2483 and cloned between *EcoR*V and *Bam*HI restriction sites of pBS-DHFR. The final construct was linearized with *Not*I prior to transfection.

Complementation of Pb*e1a_ko* by *P. falciparum BCKDH-E1a* was performed using a knock-in strategy in the promoter region of Pb*BCKDH-E1a* still present in Pb*e1a_ko* parasites. Primers 4067 and 4068 were used to amplify this promoter region and the PCR (pPbE1a) fragment was cloned between the *Not*I and *Apa*I sites of pARL-GFP-Ty-hDHFR. Primers 4070 and 4256 were used to amplify the Pf*BCKDH-E1a* cDNA (PF3D7_1312600, www.plasmodb.org) and cloned between the *Apa*I and *Nco*I of the pPbE1a-GFP-Ty-hDHFR. Finally, the 3’UTR of Pb*BCKDH-E1a* was amplified using primers 4257 and 4258 and the PCR fragment cloned between the *Nco*I and *EcoR*V sites of pPbE1a-PfE1a-Ty-hDFR in order to generate the final plasmid, pPbE1a-PfE1a-3’PbE1a-hDHFR. This plasmid was linearized by *Mfe*I prior transfection in *Pbe1a_ko* strain.

Transfections were carried out as previously described [61]. Briefly, after overnight culture of infected red blood cells (37°C, 90 rpm), mature schizont-infected RBC were purified by Nycodenz gradient and collected. 100 µL of Human T Cell Nucleofector Kit (Amaxa) and 15 µg of digested DNA. Electroporation was performed using the U33 program of the Nucleofector electroporator (Amaxa/Lonza). Electroporated parasites were mixed with blood enriched in reticulocytes from phenylhydrazine-treated mice to allow re-invasion and immediately injected (intraperitoneal) into CD1 female mice. Mice were treated with pyrimethamine in drinking water (conc. Final = 0.07 mg/ml), 24 hr after infection. After 3 drug-cycling, infected blood was collected and *P. berghei* genomic DNA was extracted with SV Wizard Genomic DNA kit (Promega). Pb*e1a_ko* pools were cloned in RAG-1 -/- mice by limiting dilution. The integration of the different constructs was confirmed by genomic PCR, using primers listed in table S2 and loss or presence of the protein was validated by Western blot analysis.

### Reticulocyte purification and invasion assay of *Plasmodium berghei*

After separation of the plasma from red blood cells and washes in RPMI by centrifugation (300× g for 10 min), mouse reticulocytes were separated from normocytes by Percoll/NaCl density gradient (1.096–1.058 g/mL) and centrifuged at 250× g for 30 min, as previously described [62], [63]. Reticulocytes were collected from the interface of the two Percoll layers and washed twice with RPMI before culturing. Invasion efficiency in normocytes or purified reticulocytes was assessed by flow cytometry as previously described [64].

### Haematocrit

Haematocrit levels were observed in mice over the course of infection with *P. berghei* wild type and Pb*e1a_ko* parasites. Briefly, every two days, haematocrit-capillaries (Hirschmann Laborgeräte, 75 mm/18 µL, ammonia heparinized 0,9 IU/capillary) were filled with tail blood and centrifuged at 10,000 rpm for 5 min to separate the blood layers. Percentage haematocrit was calculated by dividing the packed red blood cell volume length by the total blood volume length (red blood cells and serum).

### *P. berghei* phenotyping through the life cycle

The mice used for the phenotyping were pretreated with 150 µl of 6 mg/ml of phenylhydrazine and infected with 10^7^ parasites two days later. Three days after infection parasitaemia and gametocytaemia were quantified on Giemsa-stained thin blood smears. To quantify exflagellation rates 10 µl blood collected from the tail vain was mixed with 500 µl of ookinete medium (RPMI1640 containing 25 mM HEPES, 20% FCS, 100 µM xanthurenic acid, pH 7.4) and examined using an improved Neubauer hemocytometer 12 min later. The number of erythrocytes and exflagellating microgametocytes were counted and expressed as a percentage of all microgametocytes as independently determined from stained blood films.

To assess ookinete formation 100 µl infected blood were cultured in 10 ml ookinete medium at 19°C for 20 h. Live staining with a Cy3-congugated mouse monoclonal antibody against the P28 protein labeled banana shaped ookinetes and undifferentiated macrogamete derived parasites, whose shape was assessed by microscopy and recorded for at least 100 cells per sample. For transmission studies ∼200 female *A. stephensi* mosquitos were allowed to feed on the same infected mice. Exflagellation assays, ookinete cultures and mosquito feeds were performed using the same animals (three per strain) on day three of the infection.

Midguts from 20 fed mosquitoes from each cage were dissected in PBS 7 and 14 days after feeding, examined for oocysts, photographed and analysed as described [65]. On day 21 post feeding salivary glands from 20 mosquitoes per group were dissected, homogenized and sporozoites quantified in a hemocytometer. Finally, on day 23 the remaining mosquitoes were allowed to feed on uninfected mice that had been pre-treated with phenylhydrazine two days before. In parallel the salivary glands from 20 mosquitoes were homogenized and injected intravenously into different mice. All animals were observed for 14 days and the time of parasite appearance in the blood was noted.

### Metabolite extraction and analysis of *T. gondii* tachyzoites

Metabolite extraction and analysis of *T. gondii* tachyzoites was performed as previously described [20]. Intracellular and egressed tachyzoites (2×10^8^ cell equivalents) were extracted in chloroform/methanol/water (1∶3∶1 v/v containing 1 nmol *scyllo*-inositol or 5 nmol ^13^C-valine as internal standard for GC-MS and LC-MS, respectively) for 1 h at 4°C, with periodic sonication. For GC-MS analysis polar and apolar metabolites were separated by phase partitioning. Polar metabolite extracts were derivitised by methoximation and trimethylsilylation (TMS) and analysed by GC-MS as previously described [66]. For LC-MS analysis, monophasic metabolite extracts were analysed directly with a ZIC-pHILIC - Orbitrap platform and data analysed with IDEOM as previously described [19], [67]. All metabolites were identified by comparison of retention time and exact mass (LC-MS) or mass spectra (GC-MS) with authentic chemical standards, with the exception of 2-hydroxyethyl-TPP putatively identified by exact mass (<1 ppm) and predicted retention time based on a quantitative structure-retention relationship model for this chromatographic method [68].

### Stable isotope labelling of *T. gondii* tachyzoites, *P. berghei*-infected and uninfected RBCs

*T. gondii*: stable isotope labelling, metabolite extraction, and GC-MS analysis was performed for polar metabolites, as previously described [20]. Briefly, infected HFF or freshly egressed tachyzoites were incubated in low-glucose, glutamine-free DMEM, supplemented with either ^13^C-U-glucose, ^13^C-U-glutamine, ^13^C-U-leucine or ^13^C-U-leucine/^13^C-U-isoleucine/^13^C-U-valine (final concentration 8 mM, Spectra Stable Isotopes). Parasites were harvested after 4 h and metabolites extracted as above. Changes in the mass isotopologue abundances of key intermediates in central carbon metabolism were assessed by GC-MS or LC-MS analysis. The level of labelling of individual metabolites was calculated as the percent of the metabolite pool containing one or more ^13^C atoms after correction for natural abundance and amount of ^13^C-carbon source compared to ^12^C-carbon source in the culture medium (as determined by GC-MS analysis). The mass isotopologue distributions (MIDs) of individual metabolites were corrected for the occurrence of natural isotopes in both the metabolite and, in GC-MS experiments, the derivatisation reagent [69]. An untargeted metabolome-wide isotope analysis was performed on high resolution LC-MS data to detect all labelled metabolic products of ^13^C-U-glucose or ^13^C-U-leucine/^13^C-U-isoleucine/^13^C-U-valine according to the method previously described [70].

*P. berghei*: stable isotope labelling, metabolite extraction, and GC-MS analysis was adapted from the above protocol. Blood (1 mL) from individual mice infected (iRBC) with WT or *Pbe1a_ko* parasites (blood at equivalent parasitemia of ∼4%) was collected and all white cells removed by passage of the blood over cellulose CF11 columns. Parasites were cultured for maturation *in vitro* for 5 h (RPMI1640 containing 25 mM HEPES [Sigma], 0.5% Albumax, 0.2 mM hypoxanthine [pH 7.5], 25 µg/ml Gentamycin), before addition of 8 mM ^13^C-U-glucose, ^13^C-U-glutamine, or ^13^C-U-leucine as required. After 5 h of labelling, cultures were rapidly transferred to a 50 mL centrifuge tube and cellular metabolism quenched as above and schizont-infected RBCs (iRBCs) were purified from uninfected and ring-infected RBCs on Nycodenz gradient, at 4°C. iRBCs and uninfected RBCs (control) were pelleted by centrifugation (800× *g*, 10 min, 4°C), washed three times with ice-cold PBS, extracted and analysed as above.

### *In vitro* keto-acid dehydrogenase enzyme assay

Cell extracts were prepared from 10^8^ freshly egressed *T. gondii* tachyzoites by hypotonic lysis with ice-cold lysis buffer (1 mM NaHEPES (pH 7.4, Sigma), 2 mM ethylene glycol tetraacetic acid (Sigma), 2 mM dithiothreitol (Biovectra)) for 10 min on ice, in the presence of EDTA-free protease inhibitors (complete, Roche). Extracts were incubated with 0.5 mM or 2 mM of the test keto-acid (4-methyl-2-oxopentanoate, 3-methyl-2-oxobutanoate or pyruvate) in the presence of cofactors (5 mM NAD^+^, 1 mM CoA, 0.1 mM Thiamine-PP, 0.1 mM FAD and 0.05 mM lipoic acid) in 200 µL MgCl_2_/KH_2_PO_4_ (1.3 mM/33 mM) buffer (pH 7) at 37°C for 2 h (n = 2). Samples (20 µL) were extracted in 80 µL methanol/acetonitrile (1∶3) and analysed by LC-MS (pHILIC-QTOF) as described in the metabolomics methods. 3-methylbutanoyl-CoA, 2-methylpropanoyl-CoA and acetyl-CoA were quantified by reference to calibration curves of authentic standards in sample matrix (extracted lysates). Additional TPP intermediates were not quantified due to lack of authentic reference standards, and data are expressed as LC-MS peak areas. The raw high-resolution MS data were interrogated for the occurrence of other possible metabolic products derived from the branched chain keto-acids or acyl-CoAs, and none were detected. Additional controls were analysed to validate the assay including substrate-free and cofactor-free incubations, technical replicates of pooled samples for LC-MS quality control and matrix controls for RH and Tg*e1a_ko* samples (data not shown).

## Supporting Information

Figure S1 **Generation and characterization of** ***T.gondii*** **BCKDH-E1a knock-out.** (A) This scheme represents the double homologous recombination approach used to generate a direct knockout of the Tg*BCKDH-E1a*. FS, flanking sequence. (B) Genomic PCR confirming the integration in 5’ and 3’ of the HXGPRT selection cassette and loss of the cds of Tg*BCKDH-E1a* using primers indicated in panel A and Table S2.
(EPS)

Figure S2 **LC-MS analysis of acetyl-CoA biosynthesis in** ***T. gondii*** **.** Raw LC-MS high resolution mass spectra for acetyl-CoA (retention time = 13.55 min on pHILIC column) from RH and Tg*e1a_ko T. gondii* labeled with (A) ^13^C-U-glucose and (B) ^13^C-U-valine, ^13^C-U-leucine and ^13^C-U-isoleucine (BCAA). ^13^C_2_-labeled acetyl-coA (M+2.007) is only observed in ^13^C-U-glucose-labelled wild-type cells. The (M+1) and (M+3) peaks represent the natural isotopomer abundances of unlabelled (C_23_H_38_N_7_O_17_P_3_S) and ^13^C_2_-labeled (^13^C_2_C_21_H_38_N_7_O_17_P_3_S) acetyl-coA, respectively.
(EPS)

Figure S3 **Alignment of BCKDH-E1a subunits.** (A) Protein alignment comparing the Tg*BCKDH-E1a* subunit with the apicoplast Tg*PDH-E1a* of *T. gondii*. Boxed residues are the same as in (B). Amino acids boxed light blue are similar, dark blue are identical between both sequences. Tg*PDH-E1a* consists of an N-terminal apicoplast targeting sequence without homology to Tg*BCKDH-E1a*. Starting from amino acid 170 of Tg*PDH-E1a* sequence identity between both proteins is 25%. (B) Protein alignment comparing the BCKDH-E1a subunit of *T. gondii* (TGME49_039490), *P. berghei* (PBANKA_141110), *P. falciparum* (PF3D7_1312600), *P. vivax* (PVX_122460), *Homo sapiens* (P12694), *Bacillus subtillis* (P37940), *Pseudomonas putida* (P09060) and *Thermus thermophiles* (Q5SLR4). Sequences were aligned with MUSCLE 3.7 [72] and displayed with Jalview 2 [73]. The black boxed residues are the putative phosphorylation site (Ser 292 is phosphorylated in the human protein; [74]) and those in red mark an important conserved tyrosine (Tyr113 in the human protein), essential for switching between two conformational states, thereby modulating the reactivity of the ThPP cofactor [75]. (C) Tabulation of sequence identities in % of aligned BCKDH proteins in (C) starting from aa 60 of Tg*BCKDH-E1a*.
(EPS)

Figure S4 **Generation and characterization of Pb** ***BCKDH-E1a*** **knock-out strain and complementation with Pf** ***BCKDH-E1a*** **.** (A) Schematic representation of the double homologous recombination strategy used to generate Pb*e1a_ko*. The recombination event led to the replacement of *PbBCKDH-E1a* coding region with a TgDHFR selection cassette. FS, flanking sequence. (B) Parasitaemia was followed daily in mice infected with WT (blue line) or Pb*e1a_ko* (red line). Each line corresponds to the parasitaemia of one mouse. 4 mice were infected per condition. Related to Figure 4C. (C) Schematic representation of the knock-in strategy in the promoter region of Pb*BCKDH-E1a* (pPbE1a) to complement the Pb*e1a_ko* with the *P. falciparum* BCKDH-E1a subunit. FS, flanking sequence. The star represents the linearization site of the complementation plasmid containing the hDHFR selection cassette. (D) Genomic PCR analysis confirming the integration in 5’ and 3’ of TgDHFR cassette and loss of the open reading frame of Pb*BCKDH-E1a* to generate Pb*e1a_ko*. PCR analysis confirmed that the recombination event that placed the Pf*BCKDH-E1a* open reading frame under the control of the Pb*BCKDH-E1a* promoter in the Pb*e1a_ko* strain to generate Pb*e1a_ko*+Pfe1a. Primers used are indicated in panel A and C and Table S2. (E) Total cell lysates from mixed population of parasitic stages for *P. falciparum* Pf3D7, Pb wild-type, Pb*e1a_ko* and Pb*e1a_ko*+Pfe1a were analysed by western blot. Expression of BCKDH-E1a (shown by the arrow) was assessed using cross-reacting anti-PfBCKDH-E1a. Profilin was used as loading control. * represents an unspecific band, the intensity of which varied upon sample preparation. (F) Parasitaemia in mice infected with wild type (blue) or Pb*e1a_ko*+ Pfe1a (green) showing complementation of the growth defect observed in Pb*e1a_ko*. Error bars show the SD of three mice per condition.
(PDF)

Figure S5 ***P. berghei*** **, but not RBCs, catabolizes glucose and glutamine in a complete TCA cycle.** Wild type (wt) or Pb*e1a_ko* (ko) *P. berghei*-infected RBCs and uninfected RBCs (RBC) were suspended in medium containing either (A) ^13^C-U-glucose, ^13^C-U-glutamine, or (B) ^13^C-U-leucine for 5 hr. Incorporation of ^13^C into selected polar metabolites was quantified by GC-MS and levels (mol percent containing one or more ^13^C carbons) after correction for natural abundance are represented by heat plots. n.d., not detected.
(EPS)

Figure S6 **Acetate partially rescues growth defect of** ***P.berghei*** **BCKDH null mutants.** (A) Related to Fig. 6. Following *in vitro* invasion of normocytes with WT or Pb*e1a_ko*, parasites were allowed to mature *in vitro* for 20 h in media supplemented or not with 5 mM acetate. Replication of DNA content was taken as measure of parasite maturation. DNA was labelled using Vybrant DyeCycle Ruby Stain and fluorescence intensity was measured by flow cytometry. Highlighted in green is the ring/parasites degenerated early in their development fraction while trophozoite/schizont stage iRBCs are highlighted in orange. Each line corresponds to a biological replicate.
(EPS)

Table S1 **Different subunits of the BCKDH complex and the mitochondrial pyruvate carrier.**
(PDF)

Table S2 **Primers used in this study.**
(PDF)

## References

[1] Weiss LM, Dubey JP (2009) Toxoplasmosis: A history of clinical observations. Int J Parasitol 39: 895–901. 19217908 10.1016/j.ijpara.2009.02.004 PMC2704023

[2] Desai SA, Krogstad DJ, McCleskey EW (1993) A nutrient-permeable channel on the intraerythrocytic malaria parasite. Nature 362: 643–646. 7681937 10.1038/362643a0

[3] Schwab JC, Beckers CJ, Joiner KA (1994) The parasitophorous vacuole membrane surrounding intracellular *Toxoplasma gondii* functions as a molecular sieve. Proc Natl Acad Sci U S A 91: 509–513. 8290555 10.1073/pnas.91.2.509 PMC42978

[4] Jensen MD, Conley M, Helstowski LD (1983) Culture of *Plasmodium falciparum*: the role of pH, glucose, and lactate. J Parasitol 69: 1060–1067. 6371212

[5] Roth E Jr (1990) *Plasmodium falciparum* carbohydrate metabolism: a connection between host cell and parasite. Blood Cells 16: 453–460 discussion 461–456. 2257322

[6] Al-Anouti F, Tomavo S, Parmley S, Ananvoranich S (2004) The expression of lactate dehydrogenase is important for the cell cycle of *Toxoplasma gondii* . J Biol Chem 279: 52300–52311. 15459194 10.1074/jbc.M409175200

[7] van Dooren GG, Stimmler LM, McFadden GI (2006) Metabolic maps and functions of the *Plasmodium* mitochondrion. FEMS Microbiol Rev 30: 596–630. 16774588 10.1111/j.1574-6976.2006.00027.x

[8] Pomel S, Luk FC, Beckers CJ (2008) Host cell egress and invasion induce marked relocations of glycolytic enzymes in *Toxoplasma gondii* tachyzoites. PLoS Pathog 4: e1000188. 18949028 10.1371/journal.ppat.1000188 PMC2563030

[9] Sherman IW (1979) Biochemistry of *Plasmodium* (malarial parasites). Microbiol Rev 43: 453–495. 94424 10.1128/mr.43.4.453-495.1979 PMC281489

[10] Blume M, Hliscs M, Rodriguez-Contreras D, Sanchez M, Landfear S, et al (2011) A constitutive pan-hexose permease for the *Plasmodium* life cycle and transgenic models for screening of antimalarial sugar analogs. FASEB J 25: 1218–1229. 21169382 10.1096/fj.10-173278 PMC3058700

[11] Slavic K, Straschil U, Reininger L, Doerig C, Morin C, et al (2010) Life cycle studies of the hexose transporter of *Plasmodium* species and genetic validation of their essentiality. Mol Microbiol 75: 1402–1413. 20132450 10.1111/j.1365-2958.2010.07060.x PMC2859251

[12] Crawford MJ, Thomsen-Zieger N, Ray M, Schachtner J, Roos DS, et al (2006) *Toxoplasma gondii* scavenges host-derived lipoic acid despite its de novo synthesis in the apicoplast. EMBO J 25: 3214–3222. 16778769 10.1038/sj.emboj.7601189 PMC1500979

[13] Foth BJ, Stimmler LM, Handman E, Crabb BS, Hodder AN, et al (2005) The malaria parasite *Plasmodium falciparum* has only one pyruvate dehydrogenase complex, which is located in the apicoplast. Mol Microbiol 55: 39–53. 15612915 10.1111/j.1365-2958.2004.04407.x

[14] Fleige T, Fischer K, Ferguson DJ, Gross U, Bohne W (2007) Carbohydrate metabolism in the *Toxoplasma gondii* apicoplast: localization of three glycolytic isoenzymes, the single pyruvate dehydrogenase complex, and a plastid phosphate translocator. Eukaryot Cell 6: 984–996. 17449654 10.1128/EC.00061-07 PMC1951530

[15] Ralph SA (2005) Strange organelles–*Plasmodium* mitochondria lack a pyruvate dehydrogenase complex. Mol Microbiol 55: 1–4. 15612911 10.1111/j.1365-2958.2004.04314.x

[16] Seeber F, Limenitakis J, Soldati-Favre D (2008) Apicomplexan mitochondrial metabolism: a story of gains, losses and retentions. Trends Parasitol 24: 468–478. 18775675 10.1016/j.pt.2008.07.004

[17] Danne JC, Gornik SG, Macrae JI, McConville MJ, Waller RF (2013) Alveolate mitochondrial metabolic evolution: dinoflagellates force reassessment of the role of parasitism as a driver of change in apicomplexans. Mol Biol Evol 30: 123–139. 22923466 10.1093/molbev/mss205

[18] Possenti A, Fratini F, Fantozzi L, Pozio E, Dubey JP, et al (2013) Global proteomic analysis of the oocyst/sporozoite of *Toxoplasma gondii* reveals commitment to a host-independent lifestyle. BMC Genomics 14: 183. 23496850 10.1186/1471-2164-14-183 PMC3616887

[19] Limenitakis J, Oppenheim RD, Creek DJ, Foth BJ, Barrett MP, et al (2013) The 2-methylcitrate cycle is implicated in the detoxification of propionate in *Toxoplasma gondii* . Mol Microbiol 87: 894–908. 23279335 10.1111/mmi.12139 PMC3593168

[20] MacRae JI, Sheiner L, Nahid A, Tonkin C, Striepen B, et al (2012) Mitochondrial metabolism of glucose and glutamine is required for intracellular growth of *Toxoplasma gondii* . Cell Host Microbe 12: 682–692. 23159057 10.1016/j.chom.2012.09.013 PMC3990185

[21] MacRae JI, Dixon MW, Dearnley MK, Chua HH, Chambers JM, et al (2013) Mitochondrial metabolism of sexual and asexual blood stages of the malaria parasite *Plasmodium falciparum* . BMC Biol 11: 67. 23763941 10.1186/1741-7007-11-67 PMC3704724

[22] Cobbold SA, Vaughan AM, Lewis IA, Painter HJ, Camargo N, et al (2013) Kinetic flux profiling elucidates two independent acetyl-CoA biosynthetic pathways in *Plasmodium falciparum* . J Biol Chem 288: 3638–3650. 10.1074/jbc.M113.503557 PMC3868748 24163372

[23] Storm J, Sethia S, Blackburn GJ, Chokkathukalam A, Watson DG, et al (2014) Phosphoenolpyruvate Carboxylase Identified as a Key Enzyme in Erythrocytic *Plasmodium falciparum* Carbon Metabolism. PLoS Pathog 10: e1003876. 24453970 10.1371/journal.ppat.1003876 PMC3894211

[24] Fox BA, Bzik DJ (2002) De novo pyrimidine biosynthesis is required for virulence of *Toxoplasma gondii* . Nature 415: 926–929. 11859373 10.1038/415926a

[25] Hyde JE (2007) Targeting purine and pyrimidine metabolism in human apicomplexan parasites. Curr Drug Targets 8: 31–47. 17266529 10.2174/138945007779315524 PMC2720675

[26] Ke H, Morrisey JM, Ganesan SM, Painter HJ, Mather MW, et al (2011) Variation among *Plasmodium falciparum* strains in their reliance on mitochondrial electron transport chain function. Eukaryot Cell 10: 1053–1061. 21685321 10.1128/EC.05049-11 PMC3165440

[27] Boysen KE, Matuschewski K (2011) Arrested oocyst maturation in *Plasmodium* parasites lacking type II NADH:ubiquinone dehydrogenase. J Biol Chem 286: 32661–32671. 21771793 10.1074/jbc.M111.269399 PMC3173203

[28] Hino A, Hirai M, Tanaka TQ, Watanabe Y, Matsuoka H, et al (2012) Critical roles of the mitochondrial complex II in oocyst formation of rodent malaria parasite *Plasmodium berghei* . J Biochem 152: 259–268. 22628552 10.1093/jb/mvs058

[29] Srivastava IK, Rottenberg H, Vaidya AB (1997) Atovaquone, a broad spectrum antiparasitic drug, collapses mitochondrial membrane potential in a malarial parasite. J Biol Chem 272: 3961–3966. 9020100 10.1074/jbc.272.7.3961

[30] Daily JP, Scanfeld D, Pochet N, Le Roch K, Plouffe D, et al (2007) Distinct physiological states of *Plasmodium falciparum* in malaria-infected patients. Nature 450: 1091–1095. 18046333 10.1038/nature06311

[31] Sana TR, Gordon DB, Fischer SM, Tichy SE, Kitagawa N, et al (2013) Global Mass Spectrometry Based Metabolomics Profiling of Erythrocytes Infected with *Plasmodium falciparum* . PLoS One 8: e60840. 23593322 10.1371/journal.pone.0060840 PMC3621881

[32] Araujo FG, Huskinson J, Remington JS (1991) Remarkable in vitro and in vivo activities of the hydroxynaphthoquinone 566C80 against tachyzoites and tissue cysts of *Toxoplasma gondii* . Antimicrob Agents Chemother 35: 293–299. 2024964 10.1128/aac.35.2.293 PMC244994

[33] Chan XW, Wrenger C, Stahl K, Bergmann B, Winterberg M, et al (2013) Chemical and genetic validation of thiamine utilization as an antimalarial drug target. Nat Commun 4: 2060. 23804074 10.1038/ncomms3060

[34] Ævarsson A, Chuang JL, Wynn RM, Turley S, Chuang DT, et al (2000) Crystal structure of human branched-chain alpha-ketoacid dehydrogenase and the molecular basis of multienzyme complex deficiency in maple syrup urine disease. Structure 8: 277–291. 10745006 10.1016/s0969-2126(00)00105-2

[35] Huynh MH, Carruthers VB (2009) Tagging of endogenous genes in a *Toxoplasma gondii* strain lacking Ku80. Eukaryot Cell 8: 530–539. 19218426 10.1128/EC.00358-08 PMC2669203

[36] Fox BA, Ristuccia JG, Gigley JP, Bzik DJ (2009) Efficient gene replacements in *Toxoplasma gondii* strains deficient for nonhomologous end joining. Eukaryot Cell 8: 520–529. 19218423 10.1128/EC.00357-08 PMC2669201

[37] Mombaerts P, Iacomini J, Johnson RS, Herrup K, Tonegawa S, et al (1992) RAG-1-deficient mice have no mature B and T lymphocytes. Cell 68: 869–877. 1547488 10.1016/0092-8674(92)90030-g

[38] Painter HJ, Morrisey JM, Mather MW, Vaidya AB (2007) Specific role of mitochondrial electron transport in blood-stage *Plasmodium falciparum* . Nature 446: 88–91. 17330044 10.1038/nature05572

[39] Bunik VI, Degtyarev D (2008) Structure-function relationships in the 2-oxo acid dehydrogenase family: substrate-specific signatures and functional predictions for the 2-oxoglutarate dehydrogenase-like proteins. Proteins 71: 874–890. 18004749 10.1002/prot.21766

[40] Andrews FH, McLeish MJ (2012) Substrate specificity in thiamin diphosphate-dependent decarboxylases. Bioorg Chem 43: 26–36. 22245019 10.1016/j.bioorg.2011.12.001

[41] Bricker DK, Taylor EB, Schell JC, Orsak T, Boutron A, et al (2012) A mitochondrial pyruvate carrier required for pyruvate uptake in yeast, Drosophila, and humans. Science 337: 96–100. 22628558 10.1126/science.1218099 PMC3690818

[42] Herzig S, Raemy E, Montessuit S, Veuthey JL, Zamboni N, et al (2012) Identification and functional expression of the mitochondrial pyruvate carrier. Science 337: 93–96. 22628554 10.1126/science.1218530

[43] Blume M, Rodriguez-Contreras D, Landfear S, Fleige T, Soldati-Favre D, et al (2009) Host-derived glucose and its transporter in the obligate intracellular pathogen *Toxoplasma gondii* are dispensable by glutaminolysis. Proc Natl Acad Sci U S A 106: 12998–13003. 19617561 10.1073/pnas.0903831106 PMC2722337

[44] Wellen KE, Hatzivassiliou G, Sachdeva UM, Bui TV, Cross JR, et al (2009) ATP-citrate lyase links cellular metabolism to histone acetylation. Science 324: 1076–1080. 19461003 10.1126/science.1164097 PMC2746744

[45] Wang Q, Zhang Y, Yang C, Xiong H, Lin Y, et al (2010) Acetylation of metabolic enzymes coordinates carbon source utilization and metabolic flux. Science 327: 1004–1007. 20167787 10.1126/science.1179687 PMC4183141

[46] Zhao S, Xu W, Jiang W, Yu W, Lin Y, et al (2010) Regulation of cellular metabolism by protein lysine acetylation. Science 327: 1000–1004. 20167786 10.1126/science.1179689 PMC3232675

[47] Jiang W, Wang S, Xiao M, Lin Y, Zhou L, et al (2011) Acetylation regulates gluconeogenesis by promoting PEPCK1 degradation via recruiting the UBR5 ubiquitin ligase. Mol Cell 43: 33–44. 21726808 10.1016/j.molcel.2011.04.028 PMC3962309

[48] Jeffers V, Sullivan WJ Jr (2012) Lysine acetylation is widespread on proteins of diverse function and localization in the protozoan parasite *Toxoplasma gondii* . Eukaryot Cell 11: 735–742. 22544907 10.1128/EC.00088-12 PMC3370464

[49] Yamasaki M, Otsuka Y, Yamato O, Tajima M, Maede Y (2000) The cause of the predilection of *Babesia gibsoni* for reticulocytes. J Vet Med Sci 62: 737–741. 10945292 10.1292/jvms.62.737

[50] Yamasaki M, Yamato O, Hossain MA, Jeong J-R, Chang H-S, et al (2002) *Babesia gibsoni*: preferential multiplication in reticulocytes is related to the presence of mitochondria and a high concentration of adenosine 5'-triphosphate in the cells. Experimental Parasitology 102: 164–169. 12856312 10.1016/s0014-4894(03)00052-3

[51] Hall AC, Ellory JC (1986) Evidence for the presence of volume-sensitive KCl transport in 'young' human red cells. Biochim Biophys Acta 858: 317–320. 3718981 10.1016/0005-2736(86)90338-x

[52] Kirk K, Poli de Figueiredo CE, Elford BC, Ellory JC (1992) Effect of cell age on erythrocyte choline transport: implications for the increased choline permeability of malaria-infected erythrocytes. Biochem J 283 (Pt 2) 617–619. 1575704 10.1042/bj2830617 PMC1131080

[53] Liu Y, Promeneur D, Rojek A, Kumar N, Frokiaer J, et al (2007) Aquaporin 9 is the major pathway for glycerol uptake by mouse erythrocytes, with implications for malarial virulence. Proc Natl Acad Sci U S A 104: 12560–12564. 17636116 10.1073/pnas.0705313104 PMC1941508

[54] Mons B (1990) Preferential invasion of malarial merozoites into young red blood cells. Blood Cells 16: 299–312. 2257316

[55] Cogswell FB (1992) The hypnozoite and relapse in primate malaria. Clin Microbiol Rev 5: 26–35. 1735093 10.1128/cmr.5.1.26 PMC358221

[56] Plattner F, Yarovinsky F, Romero S, Didry D, Carlier MF, et al (2008) *Toxoplasma* profilin is essential for host cell invasion and TLR11-dependent induction of an interleukin-12 response. Cell Host Microbe 3: 77–87. 18312842 10.1016/j.chom.2008.01.001

[57] Soldati D, Boothroyd JC (1993) Transient transfection and expression in the obligate intracellular parasite *Toxoplasma gondii* . Science 260: 349–352. 8469986 10.1126/science.8469986

[58] Donald RG, Carter D, Ullman B, Roos DS (1996) Insertional tagging, cloning, and expression of the *Toxoplasma gondii* hypoxanthine-xanthine-guanine phosphoribosyltransferase gene. Use as a selectable marker for stable transformation. J Biol Chem 271: 14010–14019. 8662859 10.1074/jbc.271.24.14010

[59] Sinden RE, Butcher GA, Beetsma AL (2002) Maintenance of the *Plasmodium berghei* life cycle. Methods Mol Med 72: 25–40. 12125122 10.1385/1-59259-271-6:25

[60] Dessens JT, Beetsma AL, Dimopoulos G, Wengelnik K, Crisanti A, et al (1999) CTRP is essential for mosquito infection by malaria ookinetes. EMBO J 18: 6221–6227. 10562534 10.1093/emboj/18.22.6221 PMC1171685

[61] Janse CJ, Ramesar J, Waters AP (2006) High-efficiency transfection and drug selection of genetically transformed blood stages of the rodent malaria parasite *Plasmodium berghei* . Nat Protoc 1: 346–356. 17406255 10.1038/nprot.2006.53

[62] Blanc L, Liu J, Vidal M, Chasis JA, An X, et al (2009) The water channel aquaporin-1 partitions into exosomes during reticulocyte maturation: implication for the regulation of cell volume. Blood 114: 3928–3934. 19724054 10.1182/blood-2009-06-230086 PMC2773486

[63] Martin-Jaular L, Nakayasu ES, Ferrer M, Almeida IC, Del Portillo HA (2011) Exosomes from *Plasmodium yoelii*-infected reticulocytes protect mice from lethal infections. PLoS One 6: e26588. 22046311 10.1371/journal.pone.0026588 PMC3202549

[64] Pino P, Sebastian S, Kim EA, Bush E, Brochet M, et al (2012) A tetracycline-repressible transactivator system to study essential genes in malaria parasites. Cell Host Microbe 12: 824–834. 23245327 10.1016/j.chom.2012.10.016 PMC3712325

[65] Ning J, Otto TD, Pfander C, Schwach F, Brochet M, et al (2013) Comparative genomics in *Chlamydomonas* and *Plasmodium* identifies an ancient nuclear envelope protein family essential for sexual reproduction in protists, fungi, plants, and vertebrates. Genes Dev 27: 1198–1215. 23699412 10.1101/gad.212746.112 PMC3672651

[66] Saunders EC, Ng WW, Chambers JM, Ng M, Naderer T, et al (2011) Isotopomer profiling of *Leishmania mexicana* promastigotes reveals important roles for succinate fermentation and aspartate uptake in tricarboxylic acid cycle (TCA) anaplerosis, glutamate synthesis, and growth. J Biol Chem 286: 27706–27717. 21636575 10.1074/jbc.M110.213553 PMC3149361

[67] Creek DJ, Jankevics A, Burgess KE, Breitling R, Barrett MP (2012) IDEOM: an Excel interface for analysis of LC-MS-based metabolomics data. Bioinformatics 28: 1048–1049. 22308147 10.1093/bioinformatics/bts069

[68] Creek DJ, Jankevics A, Breitling R, Watson DG, Barrett MP, et al (2011) Toward global metabolomics analysis with hydrophilic interaction liquid chromatography-mass spectrometry: improved metabolite identification by retention time prediction. Anal Chem 83: 8703–8710. 21928819 10.1021/ac2021823

[69] Zamboni N, Fendt SM, Ruhl M, Sauer U (2009) (13)C-based metabolic flux analysis. Nat Protoc 4: 878–892. 19478804 10.1038/nprot.2009.58

[70] Creek DJ, Chokkathukalam A, Jankevics A, Burgess KE, Breitling R, et al (2012) Stable isotope-assisted metabolomics for network-wide metabolic pathway elucidation. Anal Chem 84: 8442–8447. 22946681 10.1021/ac3018795 PMC3472505

[71] Pino P, Foth BJ, Kwok LY, Sheiner L, Schepers R, et al (2007) Dual targeting of antioxidant and metabolic enzymes to the mitochondrion and the apicoplast of *Toxoplasma gondii* . PLoS Pathog 3: e115. 17784785 10.1371/journal.ppat.0030115 PMC1959373

[72] Edgar RC (2004) MUSCLE: multiple sequence alignment with high accuracy and high throughput. Nucleic Acids Res 32: 1792–1797. 15034147 10.1093/nar/gkh340 PMC390337

[73] Waterhouse AM, Procter JB, Martin DM, Clamp M, Barton GJ (2009) Jalview Version 2–a multiple sequence alignment editor and analysis workbench. Bioinformatics 25: 1189–1191. 19151095 10.1093/bioinformatics/btp033 PMC2672624

[74] Wynn RM, Kato M, Machius M, Chuang JL, Li J, et al (2004) Molecular mechanism for regulation of the human mitochondrial branched-chain alpha-ketoacid dehydrogenase complex by phosphorylation. Structure 12: 2185–2196. 15576032 10.1016/j.str.2004.09.013

[75] Machius M, Wynn RM, Chuang JL, Li J, Kluger R, et al (2006) A versatile conformational switch regulates reactivity in human branched-chain alpha-ketoacid dehydrogenase. Structure 14: 287–298. 16472748 10.1016/j.str.2005.10.009

