# Supplementary figures and images for: BCKDH: The Missing Link in Apicomplexan Mitochondrial Metabolism Is Required for Full Virulence of Toxoplasma gondii and Plasmodium berghei
Source: PLoS Pathog. 2014 Jul 17;10(7):e1004263. doi: 10.1371/journal.ppat.1004263 (PMC4102578; doi:10.1371/journal.ppat.1004263)

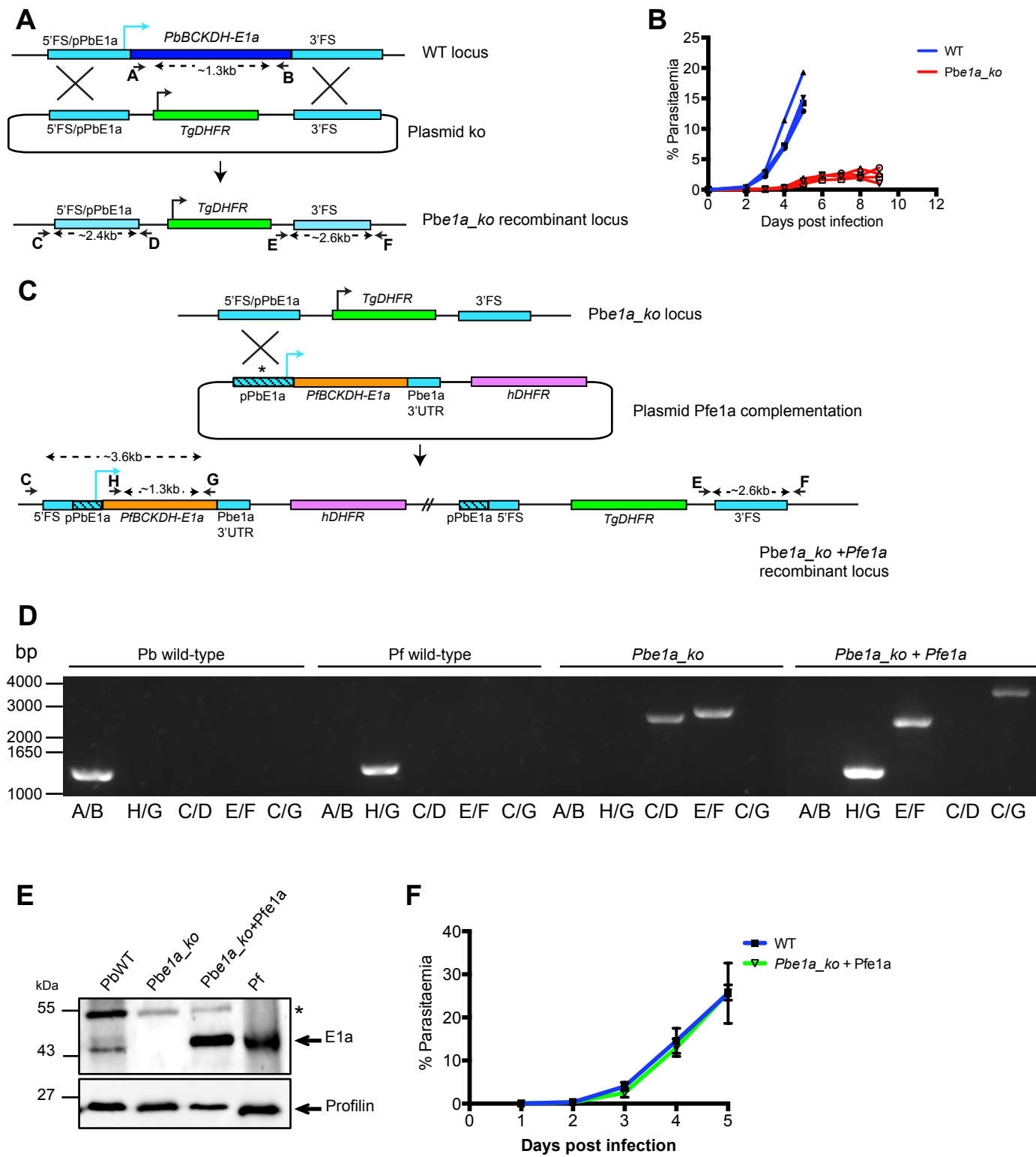

Supplement: Figure S4 — Generation and characterization of Pb BCKDH-E1a knock-out strain and complementation with Pf BCKDH-E1a . (A) Schematic representation of the double homologous recombination strategy used to generate Pbe1a_ko. The recombination event led to the replacement of PbBCKDH-E1a coding region with a TgDHFR selection cassette. FS, flanking sequence. (B) Parasitaemia was followed daily in mice infected with WT (blue line) or Pbe1a_ko (red line). Each line corresponds to the parasitaemia of one mouse. 4 mice were infected per condition. Related to Figure 4C. (C) Schematic representation of the knock-in strategy in the promoter region of PbBCKDH-E1a (pPbE1a) to complement the Pbe1a_ko with the P. falciparum BCKDH-E1a subunit. FS, flanking sequence. The star represents the linearization site of the complementation plasmid containing the hDHFR selection cassette. (D) Genomic PCR analysis confirming the integration in 5’ and 3’ of TgDHFR cassette and loss of the open reading frame of PbBCKDH-E1a to generate Pbe1a_ko. PCR analysis confirmed that the recombination event that placed the PfBCKDH-E1a open reading frame under the control of the PbBCKDH-E1a promoter in the Pbe1a_ko strain to generate Pbe1a_ko+Pfe1a. Primers used are indicated in panel A and C and Table S2. (E) Total cell lysates from mixed population of parasitic stages for P. falciparum Pf3D7, Pb wild-type, Pbe1a_ko and Pbe1a_ko+Pfe1a were analysed by western blot. Expression of BCKDH-E1a (shown by the arrow) was assessed using cross-reacting anti-PfBCKDH-E1a. Profilin was used as loading control. * represents an unspecific band, the intensity of which varied upon sample preparation. (F) Parasitaemia in mice infected with wild type (blue) or Pbe1a_ko+ Pfe1a (green) showing complementation of the growth defect observed in Pbe1a_ko. Error bars show the SD of three mice per condition. (PDF) [file ppat.1004263.s004.pdf]
