# Supplementary material for: BCKDH: The Missing Link in Apicomplexan Mitochondrial Metabolism Is Required for Full Virulence of Toxoplasma gondii and Plasmodium berghei
Source: PLoS Pathog. 2014 Jul 17;10(7):e1004263. doi: 10.1371/journal.ppat.1004263 (PMC4102578; doi:10.1371/journal.ppat.1004263)
Supplement: Table S1 — Different subunits of the BCKDH complex and the mitochondrial pyruvate carrier. (PDF) [file ppat.1004263.s007.pdf]

Table S1: Different subunits of the BCKDH complex and the mitochondrial pyruvate carrier

EC number	enzyme	*T. gondii*	*P. falciparum*	*P. berghei*
**2.6.1.42**	branched chain aminoacid aminotransferase (BCAT)	TGME49_097850	--	--
**1.2.4.4**	branched-chain alpha-ketoacid dehydrogenase E1 alpha (BCKDH-E1a)	TGME49_039490	PF3D7_1312600	PBANKA_141110
**1.2.4.4**	branched-chain alpha-keto acid dehydrogenase E1 beta (BCKDH-E1b)	TGME49_314400	PF3D7_0504600	PBANKA_110420
**2.3.1.168**	branched-chain alpha-keto acid dehydrogenase E2 (BCKDH-E2)	TGME49_119920	PF3D7_0303700	PBANKA_040230
**1.8.1.4**	lipoamide dehydrogenase E3 (BCKDH-E3)	TGME49_006470	PF3D7_1232200	PBANKA_144690
**2.7.11.4**	3-methyl-2-oxobutanoate dehydrogenase (lipoamide) kinase (BCKDK), putative	TGME49_118560	--	--
**3.1.3.52**	BCKDH phosphatase, putative	TGME49_318660	PF3D7_1309200	PBANKA_140770
	Mitochondrial pyruvate carrier subunit 1 (MPC1)*	TGME49_004370	PF3D7_1470400	PBANKA_133360
	Mitochondrial pyruvate carrier subunit 2 (MPC2)*	TGME49_035880	PF3D7_1340800	PBANKA_135420

* Both subunits were localized by IFA in the mitochondrion of *T.gondii* (data not shown)
