# Supplementary material for: BCKDH: The Missing Link in Apicomplexan Mitochondrial Metabolism Is Required for Full Virulence of Toxoplasma gondii and Plasmodium berghei
Source: PLoS Pathog. 2014 Jul 17;10(7):e1004263. doi: 10.1371/journal.ppat.1004263 (PMC4102578; doi:10.1371/journal.ppat.1004263)
Supplement: Table S2 — Primers used in this study. (PDF) [file ppat.1004263.s008.pdf]

Table S2: Primers used in this study

Name	Sequence 5’->3’	Purpose
Toxoplasma gondii		
A, 3249	CCGGGTACCTAGGATGGCGCACACGGCCTCCTC	Figure S1, Fwd at ATG to amplify Tg*BCKDH-E1a*
B, 3250	CCGACTAGTCCTGCAGGTCCTGAGAACAAGAAACGCCCACG	Figure S1, Rev to amplify Tg*BCKDH-E1a*
C, 2825	GCAGAGCTGCAATTCCACTTGCC	Figure S1, Fwd to check 5’ integration for Tg*e1a_ko*
D, 2074	CCGTAGTCTTCAATGGGTTTGGACGC	Figure S1, Rev to check 5’ integration for Tg*e1a_ko*
E, 2581	GCCCACGACAGCAGACAACTTTCC	Figure S1, Fwd to check 3’ integration for Tg*e1a_ko*
F, 2826	GTGCAGAAAGAGGGAGAAAGAGC	Figure S1, Rev to check 3’ integration for Tg*e1a_ko*
2821	GGGGTACCAGCAACCACTTAGCGACCTGAG	Fwd primer to amplify 5' flanking sequence of Tg*BCKDH-E1a* for KO in RH*ku80-ko.* With KpnI site to go in pTub5-HXGPRT-KO
2822	CCGCTCGAGGAAGCAGAAAGCGAGAGACAAGG	Rev primer to amplify 5' flanking sequence of Tg*BCKDH-E1a* for KO in RH*ku80-ko.* With XhoI site to go in pTub5-HXGPRT-KO
2823	CGCGGATCCATAAACGGAGAGGCCAGAGGAG	Fwd primer to amplify 3' flanking sequence of Tg*BCKDH-E1a* for KO in RH*ku80-ko.* With BamHI site to go in pTub5-HXGPRT-KO
2824	GTTTAGCGGCCGCGAGGTGGACTACTGGTTTAGATCC	Rev primer to amplify 3' flanking sequence of Tg*BCKDH-E1a* for KO in RH*ku80-ko.* With NotI site to go in pTub5-HXGPRT-KO
2222	CGTACGAACAAAAACTCATCTCAGAAGAGGATCTGGAAGCGTACCACGC CGGG	Fwd to clone the Cterm of BCKDH in the pTub8SOD3 vector with BsiWI site and Myc Tag.
4531	GCCTTAATTAACTACAGAGACTCGTATTCCTTCAGTTGGGG	Rev with STOP site and PacI to go in pTub8SOD3 vector create SOD3mycBCKDH fusion protein.
4540	GCCCGTACGAACAAAAACTCATCTCAGAAGAGGATCTGGCTGCGAGGTC GGGAAACCAGAC	Fwd to clone the Cterm of PDH in the pTub8SOD3 vector with BsiWI site and Myc Tag to send it to the mitochondrion.
4541	GCCTTAATTAATTAGTCAATCACAATCGGTGG	Rev with PacI to go in pTub8SOD3 vector create SOD3mycPDH fusion protein.
Plasmodium berghei		
3835	GGCGGTACCCTGTAATGTAACCATCGGTGATACG	Fwd primer to amplify 5' flanking sequence of *Pb-bckdhe1a* for KO in Pb to go in pBS-TgDHFR KpnI site
2482	GGGCCCCAAATTTTTGCAAAGTGTTCATCATG	Rev primer to amplify 5' flanking sequence of *Pb-bckdhe1a* for KO in Pb to go in pBS-TgDHFR in ApaI site
2483	GATATCGAAAATTAGTTTGAATTCCCTTGATACTG	Fwd primer to amplify 3' flanking sequence of *Pb-bckdhe1a* for KO in Pb to go in pBS-TgDHFR EcoRV site
3836	GGCGGATCCCACGATGAGGTAAAAGAGATGATCG	Rev primer to amplify 3' flanking sequence of *Pb-bckdhe1a* for KO in Pb to go in pBS-TgDHFR BamHI site
4067	CGGGCGGCCGCGTTTATAAAACCATTATTATTTATTTAACCCAAGC	Fwd primer in the promoter of Pb*BCKDH-E1a* for complementation of Pb*e1a_ko* by the PfBCKDH-E1a NotI site
4068	CGGGGGCCCCACTTTTTATTCAATTCAATACTTTTTGTTATCCC	Rev primer before ATG of PbBCKDH-E1a for complementation of Pb*e1a_ko* (ApaI site)
4257	GCCCCATGGGAATTCCCTTGATACTGTTTTTTGCACAC	Fwd to amplify 3'UTR of PbBCKDH-E1a for complementation of Pb*e1a_ko* (NcoI site)
4258	CGGGATATCCTCAGGATGACCAAGATTGTCTTGATTCC	Rev to amplify 3'UTR of PbBCKDH-E1a for complementation of Pb*e1a_ko* EcoRV site
A, 2223	GGTACCGTATGCAGGAATAATAAACTCCTAAAGTTATGTTCC	Figure S4, Fwd at ATG to amplify ORF of *PbBCKDH-E1A*
B, 2224	GGGCCCATTTTCAAATCTTGAAGTGTCATTATTATTTTTATTTTCC	Figure S4, Rev to amplify ORF of *PbBCKDH-E1A*
C, 3985	GAGGAAAAGTATAGACAACCG	Figure S4, Fwd to check 5’ integration for Pb*e1a_ko*
D, 2239	GATGTGTTATGTGATTAATTCATACACAAAC	Figure S4, Rev to check 5’ integration for Pb*e1a_ko*
E, 2238	GTCTCTTCAATGATTCATAAATAGTTGGAC	Figure S4, Fwd to check 3’ integration for Pb*e1a_ko*
F, 3986	GGAACAAAACGGACAACCTCC	Figure S4, Rev to check 3’ integration for Pb*e1a_ko*
Plasmodium falciparum		
H, 4070	GCCGGGCCCGAAAATGAGAAATATTGTTCAGAAATACTTACAAAG	Figure S4, Fwd at the ATG to amplify PfBCKDH-E1a for

		complementation of Pb*e1a_ko* (ApaI site)
G, 4256	CGGCCATGGCATCATCGCTCAAATTTTGATGTATC	Figure S4, Rev including STOP to amplify PfBCKDH-E1a for complementation of Pb*e1a_ko* (NcoI site)
2391	GGCCATGGGATTTTCTGGATACAAAATTTACAGTG	Fwd primer to amplify aa 277 to 425 of *Pf-bckdhe1a* for production of Antibodies
2392	GGGTCGACTTATCGCTCAAATTTTGATG	Rev primer to amplify aa 277 to 425 of *Pf-bckdhe1a* for production of Antibodies
